# Recent Achievements in Polymer Bio-Based Flocculants for Water Treatment

**DOI:** 10.3390/ma13183951

**Published:** 2020-09-07

**Authors:** Piotr Maćczak, Halina Kaczmarek, Marta Ziegler-Borowska

**Affiliations:** 1 Faculty of Chemistry, Nicolaus Copernicus University in Toruń, Gagarina 7, 87-100 Toruń, Poland; pmacczak@doktorant.umk.pl (P.M.); martaz@umk.pl (M.Z.-B.); 2 Water Supply and Sewage Enterprise LLC, Przemysłowa 4, 99-300 Kutno, Poland

**Keywords:** bio-based flocculants, biopolymers, polysaccharides, water treatment, flocculation mechanism

## Abstract

Polymer flocculants are used to promote solid–liquid separation processes in potable water and wastewater treatment. Recently, bio-based flocculants have received a lot of attention due to their superior advantages over conventional synthetic polymers or inorganic agents. Among natural polymers, polysaccharides show many benefits such as biodegradability, non-toxicity, ability to undergo different chemical modifications, and wide accessibility from renewable sources. The following article provides an overview of bio-based flocculants and their potential application in water treatment, which may be an indication to look for safer alternatives compared to synthetic polymers. Based on the recent literature, a new approach in searching for biopolymer flocculants sources, flocculation mechanisms, test methods, and factors affecting this process are presented. Particular attention is paid to flocculants based on starch, cellulose, chitosan, and their derivatives because they are low-cost and ecological materials, accepted in industrial practice. New trends in water treatment technology, including biosynthetic polymers, nanobioflocculants, and stimulant-responsive flocculants are also considered.

## 1. Introduction

Human activity and global industrialization are increasingly affecting the natural environment, which results in the growing pollution of natural water sources. Both groundwater and surface water can be contaminated with suspended solid particles, colloidal particles, and dissolved substances. Their removal can take place as a result of the force of gravity (this applies to larger particles), and in the case of charged particles—in the process of coagulation or flocculation (which may be independent of the surface charge). However, the most considerable difficulty is the treatment of water polluted with finely divided particles, which can be untreated sewage, heavy metal ions, and non-biodegradable pesticides or naturally occurred organic and mineral compounds [1]. Various techniques have been implemented to overcome these problems: from classical and simple methods such as sedimentation and filtration to more complex methods including ultrafiltration, ozonation and reverse osmosis, which, however, generate higher costs of the process. The techniques used in water treatment need to be specific, economical, and efficient [1,2,3].

Therefore, preferred and commonly used method is flocculation, which most frequently requires the use of particular substances—flocculants (also called flocking or clarifying agent). By using them, colloidal particles invisible to the naked eye can be removed from the water, which are not subject to gravity and cannot be effectively filtered. As a result of their very small size (the diameter of typical colloidal particles ranges from 1 nm to 1 μm) and large surface to mass ratio, in colloidal solutions, most important are surface properties and electrokinetic effects. The ionization of functional groups, ion adsorption at the particles surface, and surface charge usually depend on composition of solution and pH [4].

Flocculants—substances accelerating the agglomeration of colloidal particles and falling of floc sediments in the water system as well as increasing the removal efficiency of pollutions—are commonly used in processes of water and wastewater purification [5]. A good flocking agent is characterized by effective removal of impurities at its lowest possible concentration and in the shortest time. Previously, mainly inorganic compounds (such as aluminum sulfate and iron chloride) were used for water and wastewater treatment, owing to their high availability and low price, but currently polymers (both synthetic and natural) are increasingly popular flocculants. They are especially beneficial in increasing the rate of slow-settling aggregates at low temperatures, because of enlarging the surface area (i.e., sorption capacity) of the flocs formed [1,6,7]. However, inorganic flocculants are sensitive to pH changes and lead to large amounts of sludge in the environment. Metal ions from such sludge entering groundwater are a serious problem.

Therefore, in modern water purification technologies, polymer flocculants are increasingly used. Polymer flocculants cause the formation of large, coherent aggregates (so-called flocs) that settle in the solution. Synthetic polymers are highly effective flocculants at low dosages but have poor shear stability. In the case of water-soluble polymers, their flocculating effect depends on the size of the random coils (i.e., the radius of gyration), which are the privileged conformation in solution. However, the main disadvantages of flocculants based on synthetic polymers are the lack of biodegradability and hence environmental burden as well as the difficulty of recycling post-process sludge. Among the synthetic polymer flocculants, the most important is water-soluble polyacrylamide (PAM)—a non-ionic, amorphous polymer which can be modified to ionic form in the copolymerization process [8,9,10]. The acrylamide monomer can be used for grafting or crosslinking of other type of polymers.

Most synthetic flocculants are remarkably toxic to humans, animals, and aquatic organisms [11,12,13]. For example, acrylamide monomer, which can contaminate the polymer in trace amounts, has a dangerous carcinogenic effect [14]. It is possible that small amounts of polymers after water treatment will get into the environment in finely divided form or as diluted solution, which creates an additional problem [13]. This is the reason new biodegradable, safe, and economical substitutes of the conventional agents are sought. Therefore, the use of bio-based flocculants, which are relatively harmless to the environment, has become a common trend nowadays. 

Recently, biopolymers, particularly polysaccharides, have attracted the great attention of the scientific community mainly due to their availability, biodegradability, and high capacity to adsorb pollutants from water [3,15]. Biopolymers differ from synthetic polymers by the presence of higher-order structures and sometimes the lack of an identified repeating unit (as in the case of lignin), while they are generally characterized by a lower polydispersity or even monodispersity. On the other hand, polysaccharides are macromolecular compounds in which the repeating units are monosaccharides (glucose and fructose) linked in chains mainly by 1,4-glycosidic bonds (i.e., –C-O–C– ether bonds). Polysaccharides are biopolymers that are synthesized in nature in plants (e.g., cellulose, starch, and pectin) or in animal organisms, e.g., chitin and chitosan, the source of which are the shells of crustaceans (lobsters, crabs, and shrimp), insects, and fungi. Their properties depend on the chemical structure, rich in functional moieties, mainly hydroxyl groups but also amine, carbonyl, etc. The presence of these functional groups contributes to the effective adsorption of various pollutants in the flocculation process.

Usually, flocculants based on natural polymers are effective in high doses and are shear stable. Moreover, they can be easily modified to enhance their flocculation efficacy. According to the literature reports, the combination of properties of natural and water-soluble synthetic polymers allows the creation of new highly effective flocculants. Examples are works on the use of starch, chitosan, or cellulose and their derivatives with acrylamide in water and wastewater treatment [16,17,18]. 

The main goal of this article is to present the recent reports on natural bioflocculants application in water treatment, mainly based on polysaccharides and their derivatives or copolymers. In addition, the mechanisms of flocculation processes, methods for testing new agents of this type, and the factors influencing the purification process are briefly summarized. Particular attention is paid to new biosynthetic flocculating agents, nano(bio)flocculants, and smart (intelligent) materials. Due to the large amount of literature on flocculation in water treatment and the narrow aspects of individual published works, our intention is to summarize in one article the latest achievements on the acquisition of new biomaterials for flocculants manufacturing, research methodologies including determination of process efficacy, and future trends in this field. At the same time, the basics of flocculation mechanisms are briefly described to cover the topic fully and comprehensively.

## 2. Flocculation Mechanism

Flocculation and coagulation are the most economic methods for solid particles removal from water. However, there are some terminology mistakes between the above-mentioned processes. Flocculation is often mistakenly thought to be the same process as coagulation, but they are two different phenomena that can occur independently [19,20].

Coagulation is the process in which particles aggregate and start to form flocs that can be settled out from the water. In the first stage of coagulation, as a result of the reduction of the electrokinetic potential, the colloidal particles are destabilized. This is due to both Brownian motion leading to the collision of the particles (perikinetic aggregation) and fluid motion, in which micelles combine into larger aggregates (orthokinetic aggregation) [4,21]. During the collisions and aggregation of particles, larger and larger flocs are formed which settle out of the suspension under the action of gravity. The result is clean, colloid-free water [1,19,20,22]. Flocculation improves the conditions of the sedimentation process by joining destabilized particles together, increasing their weight, which allows them to be removed by filtration. This is an important stage of water purification, especially surface water, which removes organic impurities, including viruses and bacteria [23]. Destabilization of colloids can be achieved by the addition of electrolyte, which most often is aluminum or iron salts, in general called coagulants. Hydrolysis of these electrolytes leads to formation of colloidal hydroxides that adsorb on the surface of contamination particles present in water. According to the DLVO theory developed by Derjaguin and Landau and independently by Verwey and Overbeek [24,25], the addition of electrolyte decreases the double electrical layer until the dominant influence of attraction, van der Waals forces, occurs. This is the reason of flocs formation. Precipitation of agglomerates occurs after exceeding the critical coagulation concentration, which depends on experimental conditions (mixing, time of measurement, etc.). Usually, the settling rate is low, and, to enhance it, a small amount of organic polymeric flocculant should be added [1,26]. In industrial practice, the combination of coagulation and flocculation (described by the symbol C/F) is used by applying inorganic coagulants (electrolytes) and flocculants (ionic and non-ionic polymers). This approach contributes to the formation of larger and denser flock, and thus to faster and more effective water purification from inorganic and organic impurities [27]. The more economical version uses the non-coagulation purging process, i.e., direct flocculation. In this simplified method, cationic or anionic polymers play a dual role: neutralizing particles’ charge and aggregating them by bridging. This process is effective over a wide pH range (as opposed to coagulation) and is mainly used to remove relatively high levels of organic contaminants.

**Figure 1:**
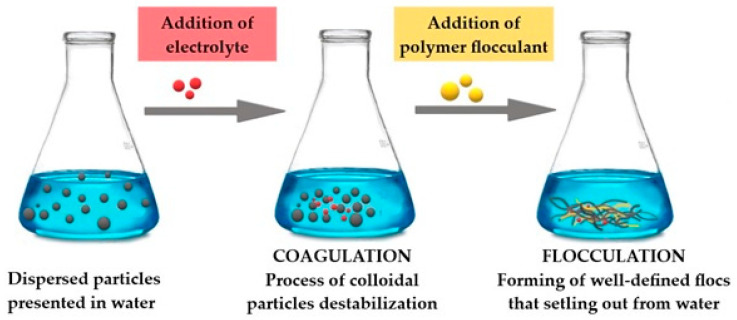
Illustration of coagulation and flocculation process.

The scheme of coagulation and flocculation is shown in Figure 1. Typically, coagulation is a very fast step (<10 s), while the flocculation is much longer (lasting 20–45 min) [21]. Moreover, aggregates growing in both processes differ significantly. When coagulating in the presence of salt, the aggregate sizes are relatively small—in this case, after a short increase in their size, a plateau is quickly reached. Flocculation with macromolecular compounds generally leads to larger aggregates, and, after reaching their maximal size, a certain decrease is observed. The restructuring of particles or irreversible breakage is responsible for this [28,29].

According to the literature reports from recent years, the flocculation mechanism in the presence of polysaccharide flocculants is considered to be due to the two main mechanisms described below: (a) charge neutralization; and (b) polymer bridging [5,6,30]. These two ways depend on the adsorption of polymer on particle surfaces as a result of electrostatic interactions, hydrogen bonding, hydrophobic interactions, complexation, or ion bridging by macromolecules [5,6,15,30]. A thorough explanation of these mechanisms should be based on detailed research at the molecular level because flocculation is a rather complicated, multi-stage process comprising several competing physical phenomena and chemical reactions [31]. Understanding these phenomena allows finding a correlation between the properties of used flocculants and the effectiveness of the flocculation process, which is important from a practical point of view.

The verification of the flocculation mechanism was presented by Lemanowicz et al. [32]. The influence of the optimal concentration of flocculant, at which flocs capable of settling are formed (this is called the flocculation window) was explained. Exceeding this concentration limit leads to the re-stabilization of the suspended particles. The influence of temperature has also been considered here, especially important when polymer properties are altered under the heat. Such polymers change the above-described mechanisms, which in this case depend of heating conditions and flocculant dose. Termo-sensitive polymers undergo not only re-conformation at a certain temperature but also their hydrophilic nature changes to hydrophobic. This alters the molecular interactions, the result of which is partially or fully reversible aggregation taking place.

A detailed description of flocculation mechanisms, supported by theoretical considerations, has recently been published in several articles [28,33,34,35,36]. Modeling of the process allowed determining the time of adsorption (τ_ads_) and aggregation (τ_agg_), which is different in Brownian diffusion and shear-induced flocculation. For example, in suspension of charged silica particles flocculated by polyacrylamide, τ_agg_ is 16 and 180 s in shear and diffusion processes, respectively [28].

The review work by Oyegbile and Ay [36] is devoted to both mechanistic and kinetic considerations. The role of physicochemical process in particle aggregation, flocs stability, molecules interactions, mechanisms of aggregate disruption, and transport processes including particles collisions in laminar and turbulent shear are also discussed here. 

### 2.1. Charge Neutralization

**Figure 2:**
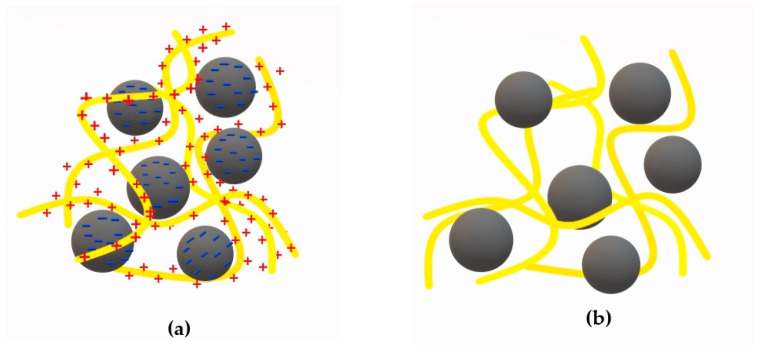
Scheme of flocculation mechanisms (the curved lines represent polymer chains adsorbing to the spherical colloidal particles): (**a**) charge neutralization; and (**b**) polymer bridging.

Charge neutralization (CN) can take place if the polymer has an opposite charge to that on the surface of the colloidal particles, as shown in Figure 2a. In this case, the particle surface charge density is reduced by adsorption of the macromolecules which results in the destabilization of this particle (repulsive electrostatic interactions are replaced by attractive forces). This mechanism is particularly effective for low molecular weight polymers (<10^5^ Da) able to adsorb and neutralize the particles suspended in water [5,37,38].

The effect of neutralizing the charge is reduction of the electrokinetic i.e., zeta (ζ) potential, which is the potential difference between dispersed particles and the medium in which they are scattered. In other words, it is the electric potential at slipping plane (shear plane), i.e., at the boundary between the compact layer and diffuse layer of particles in colloidal solution [39]. Decrease of zeta potential contributes to the creation of van der Waals’ attractive forces, facilitating aggregation and sedimentation of formed flocks [27]. 

**Figure 3:**
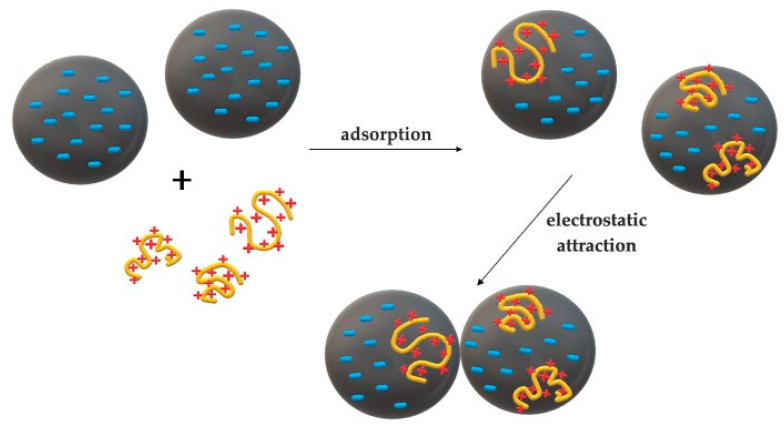
Mechanism of electrostatic patch model for flocculation (according to Bolto [6]).

A certain variation of this method is the so-called electrostatic patch model, which involves partial neutralization of the charge, which occurs in the presence of polyelectrolyte of not very high molecular weight. This process involves incomplete neutralization, thus formation of positively and negatively charged fragments on the surface of the same molecule. Such patches or “islands” with different charges cause attraction and precipitation of neighboring particles [6] (Figure 3). Flocs created in this way are more strongly bonded than in the case of ordinary charge neutralization [15,27,40].

Another mechanism described in the literature is sweep flocculation (SF) but it is also actually a different kind of charge neutralization combined with the transfer of colloidal particles to the sludge (which resembles sweeping) [4]. Initially, the negative charge of colloid particles is neutralized and then positively charged large aggregates (sweep flocks) are formed. The mutual attraction between aggregates and still present colloidal particles leads to their attachment and settling. This nonselective process occurs mainly in the presence of inorganic coagulants Al/Fe salts) and at neutral pH. In this case, sweep flocks are aggregates of Al(OH)_3_ or (Fe(OH)_3_. Other water-soluble impurities can also combine with sweep flocks or be entrapped in them. The fast SF occurs at high content of coagulants (at oversaturation). Other factors influencing this process are the presence of various anions in water and colloid concentration [4]. This type of mechanism can also take place in the presence of bio-based polymeric flocculants. 

### 2.2. Polymer Bridging

**Figure 4:**
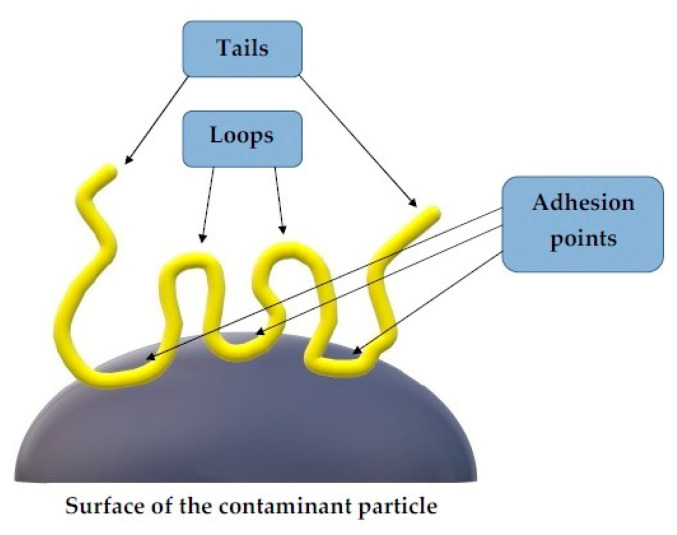
Model of polymer chain adsorbed on contamination particle (according to Bolto [6]).

In the case of bridging mechanism (Figure 4), some polymer segments are adsorbed on the surface of colloidal particles, resulting in loops and tails suspended in the solution [6], which can attach to adjacent particles to form larger aggregates—flocks (shown in Figure 2b). The polymer can be adsorbed as a result of van der Waals forces, hydrogen bond formation, or chemical reaction between the functional groups of the macromolecules and the colloidal particles. This mechanism is particularly effective for high molecular weight polymers (>10^6^ Da) having the same charge as colloidal particles [5,30]. It also applies to dispersed uncharged particles, even if they are relatively far apart (at a distance greater than the action of electrostatic attractive forces, which may occur at very low concentrations). Polymers are extremely advantageous flocculants. This is due to the entanglement of the macrochains in the form of random coils, which contribute to the entrapment of particles in their physical network, and the possibility of changing the conformation in the solution because of their high flexibility. This promotes matching the shape of the polymer chains to the surrounding or joined colloidal particles. The adsorbed macromolecules can undergo relaxation process—if they become too flat on the contamination particle surface, they are unable to combine with other particles. Similarly, too low polymer particles are inactive in bridging process [28,41]. To prevent unwanted inactivation of the polymeric flocculant, polymer mixtures are used. In this case, one polymer provides adsorption sites for the other or contributes to a more elongated conformation of adsorbed macromolecules [41].

To induce flocculation via bridging mechanism, the size of macromolecules should be larger than double the layer thickness of the colloidal particle. The estimated minimal molecular weight for linear nonionic polymer (polyacrylamide) is about 30,000 Da [28]. The aggregates formed in the presence of polymeric flocculants are stronger and greater (with size up to approximately 50 μm) than in classic coagulation by inorganic compounds. 

The undoubted advantage of applying the polymer for this purpose is the simplicity of its modification by introducing the specific functional groups able to effectively bind of impurity molecules. For example, in addition to existing hydroxyl groups in cellulose and starch, reactive carboxyl or aldehyde groups can be easily inserted to structure of macromolecules. Another polysaccharide, chitosan, containing amine and *N*-acetyl moieties, can also be used as reactive flocculant. 

Flocculation efficiency occurring according to bridging mechanism depends not only on the chemical structure and molecular weight but also on the degree of macromolecules branching. However, there are divergences in the literature on this subject. It has been published that the linear molecules promote the process effectiveness [15]. A different result was obtained in other studies on the effect of polymer architecture. Xu et al. [42] found that hyperbranched cationic polyacrylamide exhibited enhanced flocculation comparing to its linear counterpart, which has been explained by increasing interactions of branches with suspended particles. A shorter settlement time, high transmittance of purified water, and large size of precipitated flocs were observed in the presence of this novel branched polymer [42].

It should be added that, under certain, specific conditions (e.g., in very diluted solution), the polymer chain will wrap individual colloidal particles, which will restore the stable suspension. Similar effect can cause intensive mixing, during which the bridges between colloidal particles may break, especially if they arose as a result of weak dispersion forces [4]. However, in some cases, the reversibility of flocculation can be a positive aspect. It has been proved that breaking and re-growth of flocs results in a dense, compact sediment and more effective separation, even with a reduced flocculant dose [43]. It concerns all considered above mechanisms. It was found that when SF is main mechanism, the breakage and re-growth of aggregates finally leads to formation of greater flocs which begin to repulse because of the accumulated charge. In CN, smaller aggregates can fully re-grow that increase the flocculation efficiency [4,43].

This mechanism usually depends on the flocculant type: the agent with high electric charge density acts via charge neutralization while high molecular weight compounds of low content of charges flocculates mainly by bridging. If conventional C/F technology is insufficient and does not ensure high water quality standards, the enhanced coagulation (or optimized coagulation) can be applied. This process is carried out to increase the sedimentation rate and fully remove the disinfection byproduct. It can be achieved by using excess of coagulant, combination of coagulants or other additional agents (e.g., oxidant and activated carbon), adjusting pH, and controlling of hydraulic condition [44,45,46]. In addition, the sludge production is reduced and simultaneously the water treatment cost is lowered in enhanced coagulation. 

## 3. Determination of Flocculation Efficiency and Mechanisms

The basic feature indicating the presence of impurities in drinking water is turbidity; hence, the most important measuring techniques are based on determining this physical parameter. The principle of water turbidity measurement is based on the assumption that light penetrates a layer of pure water (completely transparent) in an undisturbed manner, while the presence of suspended particles causes its scattering or absorption. The degree of these disturbances (Tyndall effect) varies dependently on the size, shape, concentration, chemical composition, and refractive index of the particles.

The measurement of the intensity of the scattered or transmitted light (transmittance) is the basis of the methods used in both water treatment plants and in laboratory tests. Currently, the standard method is to measure scattered light at an angle of 90°. The instruments using this method are nephelometers (also called turbidimeters) and the measurement result is usually expressed in nephelometric turbidity units (NTU) as well as formazin turbidity units (FTU) or formazin attenuation units (FAU)—depending on the technology or method used. Apparatuses based on the measurement of transmitted light are more useful for measuring the turbidity resulting from the presence of larger particles in water (of diameter >1 µm) [47].

The new turbidimeters, instead of measuring of a single reflection at 90°, record a series of reflected beams in the full angle range (360°) around the cuvette with the sample of water tested. Recording light reflected in a full circle around the sample significantly increases the signal-to-noise ratio, which provides greater accuracy in turbidity measurement [48].

The turbidity is sometime applied for determination of color removal, e.g., in wastewater treatment from the textile industry:

(1) $$R_{d}=\frac{T_{o}-T_{f}}{T_{f}}100\%$$

where R_d_ is dye removal (%) and T_o_ and T_f_ are the turbidity of the initial wastewater and after treatment at a given time, respectively [49].

The R_d_ value is also expressed by means of determined changes in dye concentration

(2) $$R_{d}=\frac{C_{o}-C}{C}100\%$$

where C_o_ and C are the dye concentrations before and after water treatment, respectively [50].

A frequently used method for determining flocculation efficiency is the UV–Vis absorption spectroscopy. It allows determining the concentration of a given type of impurities (e.g., metal cations) absorbing in the UV–Vis range. By measuring the absorbance (or transmittance) of the initial water sample and after the purification process at a given time, the effectiveness of various flocculants, as well as the validity of the proposed methodology can be compared. For example, such parameter (η) was calculated by Vandamme [51] and Blockx et al. [52] for determination of effect of organic matter (algae) on flocculation using different flocking agents:

(3) $$\eta =\frac{{\mathrm{OD}}_{i}-{\mathrm{OD}}_{f}}{{\mathrm{OD}}_{i}}$$

where OD_i_ is optical density of the initial solution without flocculant and OD_f_ is the optical density of the same solution after flocculation process. This parameter can also be expressed as a percentage.

In the case of water turbidity, e.g., due to the presence of metal ions such as Fe^2+^ and Fe^3+^, in addition to turbidimetry, the common absorbance measurement in the range of 600–800 nm is used. After settling of particles due to sedimentation, the transmittance increases, thus the absorbance goes to zero. 

Two approaches are used to evaluate the effectiveness of flocculation by measuring turbidity or absorbance: evaluation of the appropriate solution parameters depending on the concentration of the added flocculant or the minimum settling time leading to the desired purification effect. Strictly speaking, the flocculation efficiency is higher if the settling time is shorter and the flocculant dose is lower. This last term is also called optimum dose (in the case of polymeric agent, it is optimum polymer dose, OPD), at which the lowest contamination level can be achieved. Another parameter is the removal efficiency (RE%), which can be obtained by assessing adsorption capacity at equilibrium state [53].

To evaluate the removal of fluoride, nitrate, and phosphate anions from aqueous solutions, Mohammadi et al. [54] measured the concentration of pollutants using ion chromatography techniques. The solutions were centrifuged, filtered, and injected into the chromatograph, equipped with an anionic column. The amount of the aforementioned ions adsorbed at the surface of flocculant (q_e_) was calculated as:

(4) $$q_{e}=\frac{\left(C_{0}-C_{e}\right)}{W}$$

where C_0_ and C_e_ are the initial and equilibrium concentration of the anions after adsorption (mg/L), respectively, and W is the adsorbent mass per the solvent volume (g/L).

In other works, flocculation index (FI) is proposed for process estimation [55,56]. FI is defined in different ways. For example, based on continuous monitoring flocculation by Photometric Dispersion Analyzer (PDA) [55], which allows measuring the average transmitted light intensity (dc value) and the root mean square (rms) value of the fluctuating component, FI is obtained by dividing rms by dc. In this method, FI is calculated in every second of studied process. The FI parameter reflects the size of suspended particles (the greater the degree of aggregation, the greater the FI).

Cruz et al. [56] described Continuous Flocculation Monitoring Equipment based on suspension flow meter using light of 900 nm wavelength. Two values were determined: stable component (DC) of electrical output from photodetector, corresponding to the average intensity of transmitted light, and variable component (AC) caused by a random change of the size and number of particles. FI was calculated as the ratio of the average square root value of AC and DC.

Absorption spectroscopy is also used for estimation of water shade. For example, determination of absorbance at 465 nm allows precise determination of bluish color, which cannot be assessed with the naked eye [56].

Another criterion may be the content of colloidal microparticles of specific sizes in the water suspension determined before and after treatment, which in the opinion of some authors is better than turbidity evaluation. For this purpose, microscopic techniques or even visual observation can be used. The size and shapes of flocks and their changes after addition of flocculation agent supply important information on the process course. Determining the size of the precipitated particles makes it possible to predict whether they can finally be removed in a classic filtration process. 

Sharma and co-workers [15] separated the agglomerates from supernatant by filtration, and, after drying and weighing this filtered residue, they calculated percentage content of dispersed and settled particles. Simultaneously, they measured the transmittance at 700 nm of blank and studied solutions to correlate these parameters.

The size, amount and size distribution of colloidal particles dispersed in the solution can be determined using instruments based on light scattering measurements (LS) [30]. Such measurements allow not only evaluation of flocculation efficiency, but also contribute to determining the mechanism depending on the system and conditions used.

An example is work by Zhou and Framks [31], where three cationic polymers (homopolymer of diallyldimehylammonium chloride and its two copolymers with acrylamide) of different molecular weights (1.1–3.0 × 10^5^ g/mol) and charge density (CD) (10%, 40%, and 100%) were used as flocculants for silica aggregates. They conducted experiments in apparatus constructed for aggregation (mini-thickener) and then studied the sediment particles by static LS as well as measured zeta potential of agitated suspension at 22 °C and pH 5.5. They found that mainly charge density determines the flocculation mechanism: bridging dominates at low CD (10%); electrostatic patch flocculation takes place at high CD (100%); and moderate CD (40%) causes flocculation by a complex mechanism, namely charge neutralization and bridging.

In other work, authors have studied flocs formation by an improved image analysis technique using charge couple device (CCD) camera and relevant software [55]. This method allows observing the growth of agglomerates and their destruction in the cyclic flocculation process at different shear forces. The size of flocks and their morphology can be appointed.

Since during flocculation by neutralization of electric charge (CF), the zeta potential decreases, this parameter is also a measure of the process efficiency. Based on the changes in zeta potential, not only the degree of neutralization can be determined, but also the appropriate dose of flocculant [57]. Lopez-Molando et al. [58] described zeta potential measurements in studies of semiconductor wastewater (containing cations of Sn, Pb, and Fe) treatment with the help of cationic and anionic polyelectrolytes. Based on research at different conditions, they concluded that this parameter plays key role for assessing the good coagulation–flocculation efficiency. The dependence of ζ on pH and flocculant dose was determined, and these parameters were correlated with turbidity and particle size. This research allowed designating a flocculation window for optimal dose and pH, which can be used in semiconductor industry.

The most common method in practice is the Jar Test. It is an important research tool that reflects the full scale coagulation/flocculation that can be implemented in a water treatment process. There is no simple definition of this procedure or even standard equipment. It is an experimental method whose purpose is to estimate the minimum flocculant dosage and process conditions required to achieve specific water quality. It is usually carried out in a stirring machine with 3–6 paddles and the same number of jars (beakers) simultaneously [59].

There are articles describing the modification of this method allowing for more precise and repeatable research. For example, a commercial Jar Test apparatus was modified by the addition of six turbidity meters coupled with a computerized data-acquisition system. It allowed for quantitative determination of sedimentation as a function of time [60].

Fujisaki [61] proposed a novel apparatus in which a conventional jar tester was combined with a photocouplers and a switching timer, which proved to be very useful in multiple studies [61]. In this solution photocouplers composed of 680-nm-wavelength red light-emitting diode (LED) lasers and fiber optical sensors were attached to both sides of the beaker. The measurements of turbidity by light transmission were performed at intervals in solution mixing in standardized conditions. With the help of this device, it is possible to study the kinetics, and indirectly also the mechanism of flocculation.

Another modification of the standard Jar Test, introduced by Xiao et al. [62], consisted of additional equipment with a particle image velocimetry (PIV) system. PIV is employed to monitor the particle size distribution (PSD) during flocculation process. Optical setup is equipped with laser source, high-speed CCD video camera coupling with image analysis software. Pulsed laser beam is expanded to a thin light sheet through lenses system which enable to visualize planar region of water containing suspended particles. This method is non-invasive and gives the ability to track the changes in PSD of the flocs in real time. It can be useful to characterize the flocculation dynamics, flocs strength, and their morphology [62].

Further development of this technique was presented by Smith and Friedrichs [63], who joined two methods of image analysis: the particle image velocimetry (PIV) and particle tracking velocimetry (PTV). The PTV involves tracking of individual large particles (d > 30 μm) of spherical shape, while PIV monitors the smaller particles for fluid velocity estimation. Simultaneous registration of settling velocity and two-dimensional size of particles enables very precise and automatic analysis of flocculation.

It should also be added that, to verify the effectiveness of the process and study the flocculation kinetics, adsorption measurements are performed.

## 4. Factors Affecting Flocculation

The flocculation process and its effectiveness are influenced by many factors, such as the chemical structure and properties (including charge) of both the removed substance and flocculant (in the case of polymers important is also average molecular weight and its distribution), their concentration, environment pH, ionic strength, temperature, rate of mixing, and mechanism of the process [5,57,64]. The most important factors discussed in recent studies are presented below.

### 4.1. Effect of pH

One of the most important parameters affecting flocculation efficiency is the pH of the raw water [5]. It should be remembered that pollutants, e.g., hydrolysable salts, affect the pH of water. It was found that, in an alkaline environment, increasing the flocculant dose does not contribute to improving process yield. In this case, acidification of the environment is recommended. Generally speaking, effective flocculation requires optimal pH for a given type of flocculant [65,66,67].

It has been shown that, by changing the pH from 8.5 to 12.0, 94% flocculation efficiency of *Chlorococcum* sp. microalgae can be obtained with chitosan, which is much higher than in the case of classic flocculation with aluminum sulfate and iron chloride. Residues after flocculation is suitable for further algae cultivation because it is not contaminated with metal compounds [68].

Mohammadi et al. [54] studied influence of pH on the charge of the chitosan-based adsorbent surface by determining its value in zero charge point (pH_pzc_). Zeta potential measurement helped to determine pH_pzc_ as 6.15. This means that only at this pH, the charge of the adsorbent is zero, and, at both lower and higher pH value, polymer surface becomes positively or negatively charged. In this way, it is possible to determine the optimum pH value at which the greatest efficiency of this flocculant is observed. It was pH = 3 at which the adsorbent was positively charged and could react with the anions present in the solution.

The effect of the initial pH and dose of another chitosan grafted copolymer for treating acid blue 83 (AB 83) contaminated water was investigated. [69]. Copolymer has been obtained by ultrasonic initiated grafting of CS by acrylamide and 3-acrylamide propyltrimethylammonium chloride. This new type of flocculant has been used in combination with kaolin to enhance the flocculation efficiency. The AB 83 dye removal rate get to maximum (91.9%) when the flocculant amount is 25 mg/L at optimum pH 5.0, which indicates possible charge neutralization mechanism. The improved flocculating effect in the presence of kaolin is also caused by bridging the dye and flocculant molecules.

Similar studies were carried out for tannin flocculants [70], lignin-grafted copolymers [71], and other plant-based agents [21,72]. Those works show the importance of pH, which influences zeta potential and allows to modify the mechanism’s pathway.

### 4.2. Effect of Salt 

As mentioned above, the electrolyte can play a role of coagulant which destabilize colloids and initiates the aggregation process. Particles suspended in the solution are surrounded by a double electric layer which determines their mutual repulsion and the stability of the solution. Adding electrolyte (inorganic salt) causes a reduction of the double electric layer and formation and settlement of flocs [24,25]. The introduced electrolyte contributes to a change in the ionic strength of the solution. The rate of settlement generally increases with the salt concentration. However, excess of salt can cause opposite effect. 

Impact of inorganic salts on the flocculation process depends on the ion charge. It has been found that, in the case of monovalent or divalent cations, this influence is small, but, in the presence of phosphate ions, a negative effect is revealed [73,74,75].

Several works have reported effect of salts on the viscosity and ion charge of the flocculated suspensions [73,74,75]. Investigations of seawater containing colloidal silica (0.05 wt.%) allowed explaining, along with the influences of viscosity, pH, and shear rate that of the type of electrolyte on the flocculation process [73]. The water solutions of pH 7 or 9 contained alkali and alkaline–earth metal chlorides in the concentration of 0.5 M. Cationic and anionic acrylamide copolymers were used as flocculants (dose range 0–700 g/ton of dry mass of solid). Expanded macromolecules containing anionic units change into a tangled (ball-shaped) conformation in the presence of cations. The ion adsorption capacity of the silica and the polymer flocculant exhibit inverse order. Electrolytes prevent interactions with silica in the presence of cationic polymer, whereas anionic flocculant exhibits strong interactions leading to formation of agglomerates and three-dimensional network, responsible for higher viscosity. The effect of ions on local water structure in studied systems has also been thoroughly discussed. Mg^2+^ ions strongly shielded the silica particles, increasing hydration sphere and weakening of the repulsion between silica and anionic flocculant particles, contrary to K^+^ and Na^+^. These studies provide information on the processes involved in desalination of seawater.

Rapid aggregation and deposition of flocs in the presence of electrolyte is also used in the treatment of flotation waste.

### 4.3. Effect of Shear Rate

The stability of flocs, necessary for their settlement, depends on the strength and number of interfacial interactions between agglomerated particles. If there are only weak and sparse of contact points, the flocs can easily break into smaller and separated parts. One factor for the disintegration of the flocs is intensive mixing with high shear forces [76,77,78].

It has been found that increase in shear rate causes an efficient fragmentation and erosion of flocs [62]. However, the consequence of this process can be reconnection (re-growth), in which smaller flocs join together when the shear forces are reduced again. Thus, the re-flocculation takes place. The flocs disintegrate and grow simultaneously until a steady state is reached [36].

Breakage of flocs can be fully or partially reversible process dependently on the type of the flocking agent used. In addition, the three-dimensional networks of silica particles and acrylamide flocculant formed in the presence of electrolytes worsen its stability with increasing shear rate [73].

Because the strength of precipitated agglomerates, dependent on shear rate, is responsible for flocculation efficacy, it is important to properly design and construct flocculation reactors for water purification technology [36].

### 4.4. Effect of Other Factors

An important factor having a significant impact on the flocculation process is the concentration, or strictly speaking optimal dose of flocculant, which is outlined in Section 3. Both an insufficient and too high concentration make the process ineffective. Another factor is the appropriate flocculation time, which depends on the type and amount of impurities in the solution and the kind of flocculant.

Since the size, shape, density, and speed of sediment settlement change over time, the changing hydrodynamics of sediments also affects the course of flocculation [76,77].

Moreover, the degree of turbidity, which depends on the type and size of suspended particles in water, can affect the effectiveness of water purification. Sometimes higher turbidity is easier to remove even with a small dose of flocculant, due to the high probability of particles collision, while smaller particles or more diluted solutions exhibiting less turbidity may be more difficult to remediate. 

Finally, the effect of temperature should be mentioned. Generally, chemical reactions and physical processes occurring at lower temperatures are slower but significant difference in flocculation can be observed only with large temperature differences [1]. Increasing the temperature accelerates the movement of molecules in the solution, increasing the probability of their collisions and aggregation. Obviously, a significant temperature effect is observed when using thermo-responsive flocculants [28,32]. 

## 5. Bio-Based Polysaccharide Flocculants for Water Treatment

The typical substances that support the flocculation process are polyelectrolytes, which are high molecular weight organic polymers. Among the wide range of natural polymers, polysaccharides have received unflagging popularity as bio-based flocculants. Those kinds of compounds are particularly attractive in water and wastewater treatment through their many advantages, such as biodegradability, accessibility, and structural features facilitating their chemical modification. These features make polysaccharides relevant agents for removal of turbidity, COD (chemical oxygen demand), microorganisms, and many other pollutants present in water [5,38,79,80,81]. The polysaccharides flocculants based on starch, chitosan, cellulose, and their derivatives are listed in Table 1.

Table 1 presents examples of bioflocculants described in recent literature and their possible applications with appropriate references. As can be seen, the cited substances (compounds and macromolecules) are mainly used to remove turbidity, metal cations, inorganic anions, dyes, pesticides, minerals, and biological contaminations. More detailed characterization of main bio-based flocculants is presented in proceeding subsections.

### 5.1. Starch and Its Derivatives

**Figure 5:**
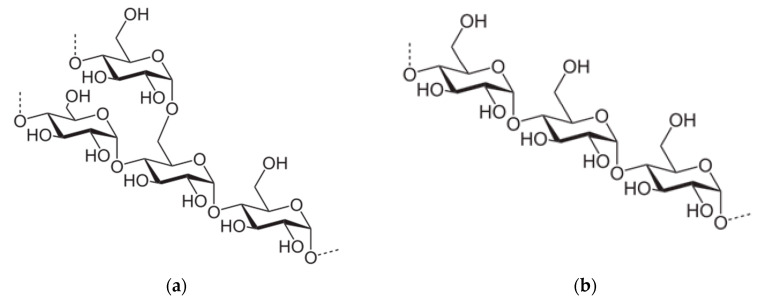
Chemical structure of starch components: amylopectin (**a**); and amylose (**b**).

Starch is a well-known biopolymer that is made of glucose units [130]. It is the most important plant reserve material and one of the most common polysaccharides extracted mainly from potatoes, corn, wheat, or rice. It is composed of water-insoluble amylopectine (poly-*α*-1,4-D-glucopyranoside and *α*-1,6-D-glucopyranoside)–an amorphous polymer, and amylose (poly-*α*-1,4-D-glucopyranoside), a semi-crystalline polymer, which can form colloidal solutions in water. The amylose is organized into straight chains of glucose residues linked by 1,4-glycosidic bonds, while amylopectin creates multiple branched chains [130,131]. The starch components are shown in Figure 5.

Different forms of starch can be used in the flocculation process [82,83,132]. Most often, the studies concern starch derivatives modified for increasing their flocculating activity [15,82]. It can be achieved by copolymerization and introduction of ionic groups. Industrial cationic starches have shown good flocculation ability, which predisposes them to remove aluminosilicate suspensions, especially natural clays from water [82,83].

Starch for flocculation was modified in graft co-polymerization [16,84,85,86,87]. Li et al. [84] prepared two kinds of starch-based flocculants, (2-hydroxypropyl)trimethylammonium chloride (CTA) and etherified carboxymethyl starch (CMS), with a different ratio of the substituent to initial polymer. The studies revealed that the macromolecule with a higher amount of CTA had a positive surface charge (CMS-CTA-P), while the other one with less substitution degree (CMS-CTA-N) was negatively charged. In the experiment, the authors used kaolin and hematite suspensions as synthetic wastewater and studied flocculation performance of both oppositely charged starch derivatives in various pH. It was found that the best efficiency is observed when the starch flocculant has opposite charge to those of the contaminations. 

These results prove that the mechanism of the observed process in this case is complex and consists in neutralization of the charge (which dominates) and a partial share of bridging.

In other reports, starch derivatives were prepared by microwave-assisted modification. In this technique, the microwave heating (conducted in a simple microwave oven) induces changes in properties of starch, mainly in solubility and viscosity [133,134,135]. Mishra et al. [16] proposed graft copolymerization of starch (St) and polyacrylamide (PAM) in the presence of ceric ammonium nitrate as an initiator (generating free radicals under microwave radiations). The obtained grafted starch (St-g-PAM) exhibited a higher intrinsic viscosity of the polymer, which increased flocculation efficacy. Moreover, applied synthesis method was fast, reliable, reproducible, and led to higher quality of copolymer comparing to obtained in the absence of microwaves.

Another approach was to evaluate flocculation efficiency of cationic starch modified with 2,3-epoxypropyltrimethylammonium chloride [83]. Investigations were conducted on model suspensions of aluminum silicates, bentonite, and natural clay. The turbidity reduction of the prepared solutions over time was analyzed. The obtained results show that the studied material effectively accelerated sedimentation of impurities. Despite the reports that the dosage has to be 2–4 times higher (2–4 mg/dm^3^) than in the case of conventional polyacrylamide flocculants to achieve similar or better results, it can be concluded that starch-based compounds can be considered as a potentially good alternative for synthetic polymers.

It should be noted that starch designed for flocculants can be obtained from various plant sources, sometimes locally available, e.g., sago, which is extracted from the tropical palm stems [136].

### 5.2. Chitosan and Its Derivatives

**Figure 6:**
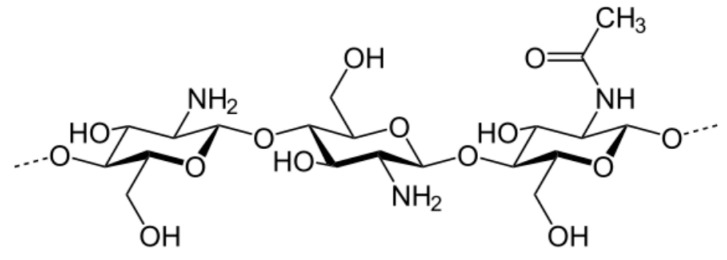
Chemical formula of chitosan—copolymer of d-glucosamine and *N*-acetyl-d-glucosamine.

Chitosan (CS) is another important polysaccharide used in flocculation. It is a deacetylated derivative of chitin, which is a component of crustacean shells (crabs, lobsters, shrimps, etc.) and cell walls of fungi. This polysaccharide consists of a linear copolymer of d-glucosamine and *N*-acetyl-d-glucosamine (Figure 6) [137]. It is highly regarded for use in water or wastewater treatment due to its reactive amino and hydroxyl functional groups, which can react with impurity particles [3,17,138,139,140]. It was stated that the main mechanism emerging during flocculation with chitosan participation is bridging [128]. Flocculation performance, similar to other properties of chitosan, depends on its deacetylation degree as well as pH of medium (pK_a_ of CS is ~6.5). Chitosan is soluble in acidic solution in which the amino groups are protonated but insoluble in neutral and alkaline environments.

As a result of its chemical structure and complexing ability, chitosan has a high affinity for many dye classes, and it can be a universal sorbent of metals and surfactants as well as microalgae [140,141]. However, similarly to starch, it is insoluble in water, and it must be modified to increase its applicability and flocculating activity. There are only a few reports [5,88,89] with the use of this polymer in its unmodified form. Al-Manhel et al. [89] studied application of chitosan dissolved in acetic acid in purification of water from the local river. The obtained results show a high flocculation efficiency of chitosan, confirmed by the reduction of water parameters such as turbidity or total dissolved solids (TDS). Similar studies were carried out by Pontius [86], who made a series of Jar Tests on surface water samples, comparing coagulation/flocculation ability of chitosan with commonly used inorganic coagulants (aluminum sulfate and iron chloride). Studies have confirmed that, as the polysaccharide concentration increased, the turbidity of water decreased, which was the basic indicator of the effectiveness of this biopolymer used. However, the capability of water purification by chitosan was lower than for commercial coagulants. The polysaccharide removed about 68.9% turbidity while aluminum sulfate and iron chloride allowed a reduction of more than 95%. In addition, in both the above-mentioned works, the dose of chitosan was quite high, which did not allow obtaining the values of the determined parameters in accordance with the high standards for drinking water. 

For this reason, attempts were made to combine the properties of chitosan with synthetic polymers to create a biodegradable and more effective flocculants. For example, Tao et al. received a water-soluble chitosan-acrylamide-fulvic acid (CAMFA) terpolymer [37]. Flocculation tests were conducted on model dyes solutions of Reactive black 5 (Rb-5), Acid blue 113 (Ab-113) and methyl orange (MO). The measure of flocculation efficiency was the water discoloration. The obtained results indicate the high efficiency of CAMFA terpolymer in removing Rb-5 and Ab-113 dyes reaching over 90%, although in a very high dose of up to nearly 300 mg/L. The effectiveness was observed over a wide pH range.

Applications of copolymers of grafted chitosan are increasingly proposed in the wastewater treatment process [5,27,38]. Based on the assumption that flocculation properties can be improved by introduction of functional groups into macromolecule structure, Wang et al. [90] synthesized a series of graft chitosan flocculants. They demonstrated that high content of methacrylate ethyl trimethyl ammonium chloride (DMC) groups results in better flocculation ability than acrylamide grafted polymer. The combination of properties of chitosan and DMC monomer increased number of positive charges that can neutralize the opposite charges presents on the surface of the particles suspended in water. 

The next type of modified chitosan flocculant was synthesized by reaction of carboxymethyl chitosan (Chito-CTA) with quaternary ammonium reagent. The amphoteric chitosan-based polymer was prepared by adding 3-chloro-2-hydroxypropyl trimethyl ammonium chloride to the Chito-CTA solution, which improves polymer solubility and increases its applicability as flocculant in water treatment [92].

Examples of novel, environmentally friendly flocculants were described by Sun and coworkers [95]. They obtained grafted copolymers of chitosan and acrylamide or [2-(acryloyloxy)ethyl] trimethylammonium chloride by UV-initiated copolymerization. The obtained materials were characterized by a porous structure, which resulted in better flocculation efficiency during water purification from zinc phosphate (removal of approximately 99%).

Another proposition by Sun et al. [94] was chitosan-based copolymers with xanthate and sulfonic acid group. This agent, also obtained in photochemical reaction of carboxylated chitosan, proved to be very effective in purifying water from heavy metal ions such Cr and Ni (total removal efficiency in both cases exceeds 99%). In this work, molecular interactions at the interface (at microscale) were studied. Moreover, the relationship between flocculant chemical structure and flocculation performance was established using FTIR, NMR, XRD and SEM analysis. 

In other work, copolymerization of the chitosan with acrylamide (AM) and 3-acrylamide propyltrimethylammonium chloride was initiated by ultrasonic waves. Flocculant (CTS-g-PAA) obtained by this way was used to remediate water from acid blue 83 (AB 83) [69]. During ultrasonic initiation, the cavitation phenomenon occurs. It is based on the formation and rapid disappearance of gas bubbles in the liquid, which is accompanied by sudden pressure changes allowing the release of a high amount of energy. It can increase the production of free radicals from initiator and thus initiate the copolymerization reaction. According to this research, the copolymer flocculant was able to remove the AB 83 dye in nearly 80% yield at optimum dose of 25 mg/L. Addition of kaolin particles to the solution improved flocculation efficiency to 91.9% by enlarging the surface area of flocs adsorbed by the CTS-g-PAA.

Recently, it was stated that combination of properties of chitosan and starch leads to promising results in the wastewater treatment. The synthesis and properties of novel flocculant based on cationic starch/chitosan crosslinking-copolymer (CATCS) was reported by You and coworkers [142]. Based on studies in kaolin suspension (at 5 g/L concentration), they proved that CATCS exhibits better flocculation properties in both acidic and alkaline environment than cationic starch and chitosan applied separately.

Chitosan is proposed to coagulate pollutants in wastewater generated in the production of chitin. Tran’s idea [93] was a two-step process: initial sedimentation at pH range 4–11 (during which turbidity was already reduced by 80%), followed by coagulation with chitosan (total removing turbidity 99.4% at pH 10.6 and dose of 86.4 mg/L). It is worth emphasizing that the residue recovered by coagulation is rich in protein (55 mg/g) and can be used as supplement in animal feed or plant fertilizer. 

Wei et al. developed a water-soluble chitosan derivative which found application in removing of dye (reactive brilliant red) from the wastewater of the textile industry [143]. The modification of chitosan was based on the etherification reaction with cationic agent (2,4-bis(dimethylamino)-6-chloro-[1,3,5]-triazin). The authors pointed out that, unfortunately, the wastewater treatment process produces a large amount of sludge harmful to the environment. On the other hand, textile dyeing effluents often contain azo dyes, which can be a valuable raw material. Recovery of these compounds has proved to be very profitable. After release from flocs, they can be used for production of nitrogen-doped carbon materials through carbonization. The proposed method decreases the amount of toxic substances in the solid post-process residues. Simultaneously, obtained material can find practical application as super-capacitor due to high electrochemical capacitance and good, long stability.

Continuing their research, this team used chitosan-containing textile sludge as precursor of graphene-like carbon nano-sheets designed as electrode material for supercapacitors [144]. This work focuses on controlling desorption of azo-dye by adjusting pH. Thereafter, the product was subjected pyrolysis in the presence of Fe (III) salt as graphitization catalyst.

Although the disadvantage of a chitosan is insolubility at pH ≥ 7, its role as a flocculant is steadily growing. There is scant information in the literature on other raw materials of animal origin—an example may be isinglass from the shredded fish bladders [21].

It should be mentioned that unmodified chitin was also used as a flocculant. It turned out that chitin is not inferior to the quality of aluminum sulfate as a coagulant. Moreover, it is stable at all pH ranges [136]. The possibility of using chitin is advantageous from an ecological point of view because it includes the management of seafood waste, and also does not require the use of chemical treatment, i.e., reagents (inorganic acids and bases) that burden the environment, as in the case of chitosan obtaining.

### 5.3. Cellulose and Its Derivatives

**Figure 7:**
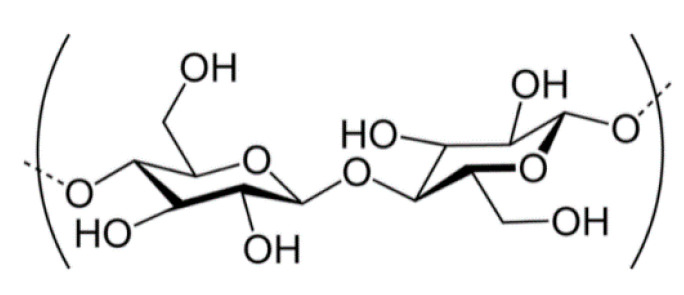
Chemical structure of cellulose unit.

Another example of valuable bio-based flocculant is cellulose which is linear polymer consisting of D-glucose molecules linked by *β*-1,4-glicosydic bonds (Figure 7). Native cellulose has a regular hydrogen bonds network that determines its mechanical strength and other physicochemical properties. 

This polysaccharide mainly forms type I (in which two modifications can occur—I_α_ and I_β_ with triclinic and monoclinic unit cells, respectively). Other types are also distinguished: cellulose II (most stable), III, and IV, differing in the organization of macromolecules [145,146].

Most often, cellulose in a chemically modified form is proposed as a flocculant (e.g., as a copolymer). Okieimen et al. [18] determined the flocculation characteristic of carboxymethyl cellulose-*g*-polyacrylamide copolymer with kaolin suspension. The studies showed that graft copolymers of cellulose can be an effective flocculant satisfactorily reducing water turbidity. According to numerous reports, this polysaccharide can be considered as a good material to produce environmentally friendly flocculants because of its physical characteristics, chemical reactivity, and flocculation efficacy [5,92,147,148].

An important reason for the necessity to modify cellulose is its insolubility. The transformation of cellulose into a more useful soluble form is possible by chemical reactions leading to ionic character. There are numerous studies on cellulose derivatives of anionic [49,98,99,102], cationic [49,103,149] and amphoteric [104] nature, as high-performance polymer flocculants.

The flocculation efficiency of anionic cellulose (dicarboxylic acid cellulose, DCC) was examined in the coagulation–flocculation treatment of municipal wastewater. DCC in the form of nanofibrills was obtained by cellulose oxidation with periodate and chlorite. It has been demonstrated a good flocculation performance, similar to that of the commercial synthetic flocculant, resulting from the high charge density and high nanofibril content [98]. This flocculant lowers significantly residual turbidity and COD. Moreover, it has proved to be very stable in aqueous solutions during long-term storage.

In the same group, three other anionic sulfonated nanocellulose flocculants (ADAC) with variable charge density were synthesized [102]. Particular attention was paid to the morphology and strength of the flocs as observed by an optical monitoring device (MOFI). It was found that ADAC can be successfully used in lower doses (compared to the inorganic agent iron sulfite). The flocs formed are smaller, rounder, and more shear stable than those formed in the presence of conventional polymer flocculant. 

In another study, it was found that oxidized cellulose, or more specifically dicarboxylic acid nanocellulose, removes 99.5% of turbidity from an aqueous kaolin suspension [99].

An example of useful water-soluble cellulose is its quaternized derivative [103]. This cationic flocculant, obtained from native cellulose by reaction with 2,3-epoxypropyltrimethylammonium chloride (EPTMAC) as an etherification reagent, was very effective in removing model anionic dyes. The advantages of quaternized cellulose derivatives are their biodegradability, a simple way of regeneration with NaOH and acetone, and the possibility of reuse. The effectiveness of this flocculant was dependent mainly on substitution degree of cellulose but not influenced by temperature and pH.

Grenda and co-workers [49] described the chemical modification of cellulose leading to cationic and anionic polyelectrolytes with different charges. The raw materials for obtaining flocculants were *Eucalyptus* bleached pulp and a cellulosic pulp with high lignin content (∼4.5 wt%). Anionic cellulose was prepared by oxidation with sodium periodate (NaIO_4_) or sodium metabisulfite (Na_2_S_2_O_5_), while cationic polysaccharide was obtained in reaction with Girard’s reagent (betaine hydrazide hydrochloride, C_5_H_14_ClN_3_O). Modified cellulosic materials were used for purification of colored wastewater from textile industry. Additionally, complexation agent–bentonite was added to the solution to promote dye adsorption (it is removed from the water together with the adsorbed dye during flocculation). Several experimental techniques were used to characterize these cellulose-based polyelectrolyes and monitor the flocculation process (among others laser diffraction spectroscopy (LDS), dynamic light scattering (DLS) and electrophoretic light scattering (ELS)). The comparison of the flocculation results of the compounds tested with the commercial polyacrylamide flocculants shows their great suitability for water treatment.

An amphoteric flocculant was obtained by grafting of methacryloxyethyltrimethyl ammonium chloride (DMC) and acrylic acid (AA) copolymer onto microcrystalline cellulose [104]. Synthesis has been conducted in solution of NaOH with urea in optimized conditions. It has been established that the best properties showed cellulose with 0.52 and 1.01 degree of substitution for AA and DMC, respectively. The maximal turbidity removal (99.82%) for kaolin suspension was found for dose of 5.0 g/L at neutral pH. Moreover, a synergistic effect was also found when using this grafted cellulose and polyaluminum chloride. Combining flocculation and ozonation was successful in both decolorization and turbidity removal in wastewater from the paper industry.

Application of cellulose and its derivatives in wastewater treatment of petroleum industry was recently reviewed by Peng et al. [105]. In this case, water can be polluted at every stage of the oil processing by organic and inorganic substance as well as suspended solid particles. Cellulose-based materials play a dual role—superadsorbent (important in removing spilled oil) and flocculant—involved mainly in the removal of colloidal particles. Among the advantages of these cellulosic flocculants are the availability of raw material, cheap production, low energy consumption and environmental protection.

### 5.4. Other Examples of Natural Polymers Flocculants

In addition to those mentioned above, some other natural polysaccharides such as sodium alginate and xanthan have recently been considered as potential bioflocculants. To increase their flocculation efficiency, various synthetic monomers were grafted onto these biopolymers.

**Figure 8:**
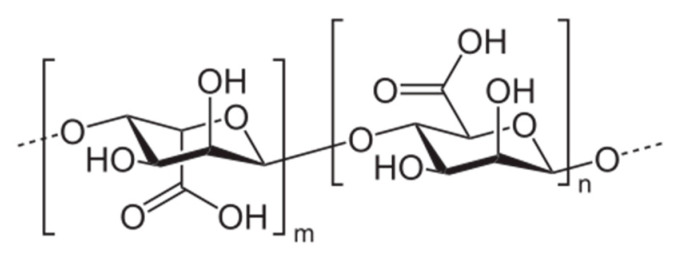
Chemical structure of alginic acid (carboxyl groups convert to carboxylate as pH increases).

Alginic acid (Figure 8) salt—sodium alginate (SA)—is gaining popularity due to its biodegradability and relatively high content of carboxylic groups, which are responsible for the ability to adsorb heavy metal ions from water [108]. To enhance its flocculating action, Tian and co-workers [108] prepared an amphoteric alginate flocculant (SA-CTA) by combination of the polysaccharide with 3-chloro-2-hydroxypropyltrimethyl ammonium chloride (CTA). As a consequence of the presence of both cationic –N^+^(CH_3_)_3_ and anionic –COO– groups, the macromolecules of SA-CTA have an ability to flocculate heavy metal ions (Pb^2+^), as well as negatively charged humic acids contaminating water.

Zhao and coworkers [109] proposed another modification of polysaccharide using microwave assisted copolymerization, in which the flocculant sodium alginate-dimethyl diallyl ammonium chloride was received. The reaction was carried out in microwave conditions with the optimal exposure time of 18 min, during which free radicals were efficiently formed and the acceleration of copolymerization was observed. It was found that the obtained copolymer effectively decolorizes water.

**Figure 9:**
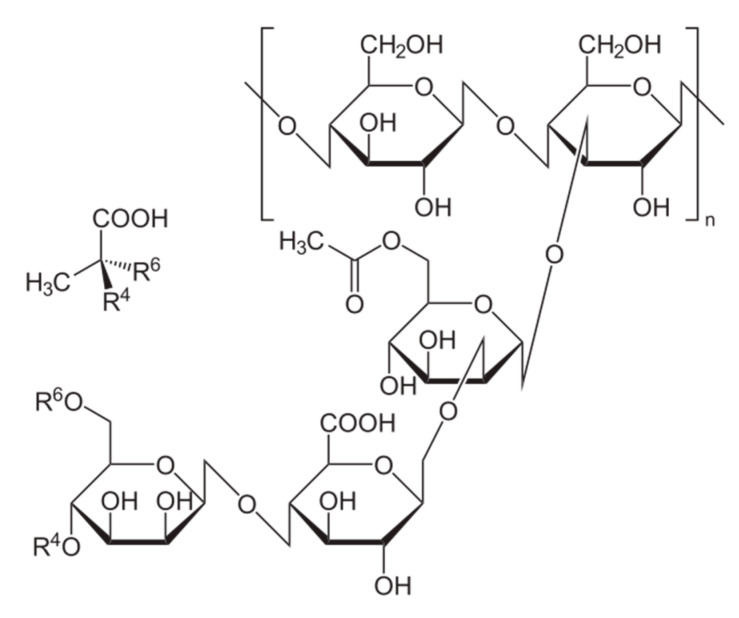
Chemical structure of xanthan.

Xanthan and other natural gums have also been proposed as a safer alternative to the commercial flocculants in water and wastewater treatment. This group of hydrocolloids, obtained by fermentation of carbohydrates by *Xanthomonas campestris*, is made of glucose, mannose, and glucuronic acid as well as partially esterified acetic and pyruvic acids (Figure 9).

Polyacrylamide grafted xanthan gum/silica (XG-g-PAM/SiO_2_) were investigated in kaolin and iron ore suspensions. Grafting was free radical process in inert atmosphere. Silica nanoparticles were incorporated to graft copolymer by hydrolysis and condensation of tetraethylorthosilicate (TEOS). Study of flocculation properties of these new hybrid nanocomposites showed the relationship between dosage and the turbidity of effluent. The increasing of flocculant dose with optimum polymer concentration of 2.0–2.5 ppm results in reduction of tested wastewater turbidity because more particles were able to bridge together and form well-defined flocs. The results indicate that that modified xanthan gum could be an efficient flocculant operating by bridging mechanism [113]. Previous work devoted to this nanocomposite revealed the excellent efficiency in removing of Pb^2+^ ions due to high hydrodynamic radius and volume of macromolecules in nanocomposite. Moreover, this adsorbent showed good recyclability [114].

**Figure 10:**
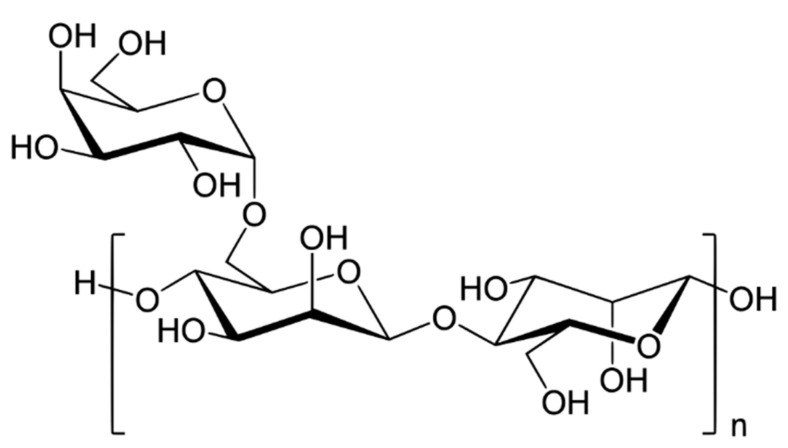
Chemical structure of the guar gum unit.

Another example of those kind compounds used in water treatment is the guar gum and their derivatives [124]. Guar gum is an organic compound belonging to the galactomannan group, i.e., polysaccharides whose chains are built from mannose units with monogalactose side branches (Figure 10). The natural polymer was cationized by the connection with glycidyltrimethylammonium chloride. Flocculant formed in this way was able to create high-density bentonite aggregates, which enabled their rapid sedimentation [125]. 

Other bioflocculants based on dextran [116,117], pullulan [110,111,112], or pectin [118] have also been reported. Most of these polymers and their grafted derivatives have promising flocculating ability in reducing color, turbidity, COD, or heavy metal ions in various types of wastewater. However, these reports are only from laboratory flocculation tests and mostly carried out on model compounds, so they are not commercially applicable so far. 

Recently, the reports describing the possibility of using lignin as a flocculant were published [50,126,150]. 

**Figure 11:**
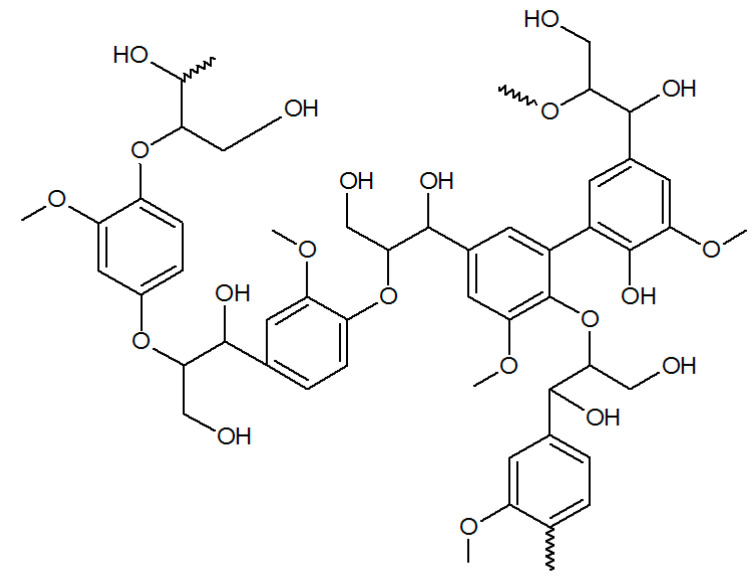
Chemical structure of a lignin fragment.

Lignin is a biopolymer that is part of wood with a complicated, crosslinked structure, containing aromatic rings, ether, and hydroxyl groups, mainly phenolic (Figure 11). To obtain adequate efficiency, it undergoes modification, e.g., oxidation or sulfomethylation [50]. In this way, a negatively charged lignin is obtained, making it suitable for removing cationic dyes from water. It has been proved that modified kraft lignin (which is the main byproduct of the Kraft pulping–sulfate conversion of wood into pulp) formed complexes with dye, which undergo fast precipitation.

Modification of lignin by polymerization with 2-[(methacryloyloxy)ethyl] trimethylammonium chloride (DMC) was described by Hasan et al. [126]. Five cationic water-soluble polymers with different molecular weights and charge densities were obtained and then applied as flocculant for kaolin suspension. It was found that the best flocculation properties showed polymer with highest charge density and molecular weight.

The effect of charge density and molecular weight of flocculants derived from paper industry sludge on the water treatment efficiency was studied by Guo and co-workers [151]. The structure and properties of chemically modified flocculants have been studied by infrared spectroscopy, gel permeation chromatography, X-ray photoelectron spectroscopy, and particle charge density determination. These compounds were applied in decontamination of water solutions containing reactive blue dye. It has been proved that the efficiency of discoloration was mainly dependent on charge density, however, the effect of molecular weight was ambiguous.

**Figure 12:**
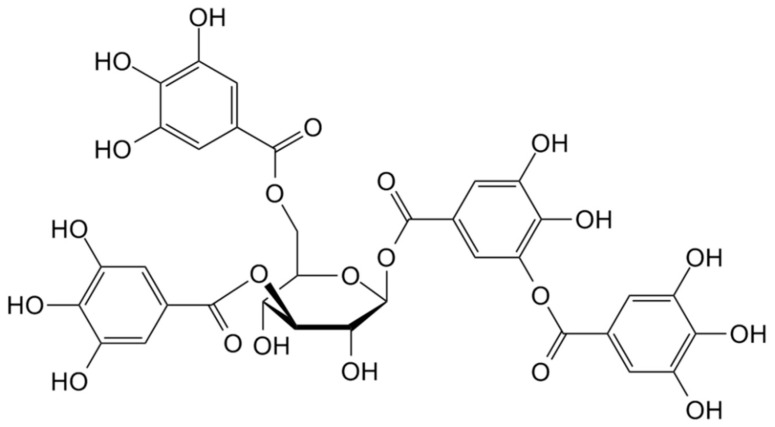
Chemical structure of tannic acid, a type of tannin.

Tannin (Figure 12)—a natural polyelectrolyte of polyphenol type (containing also esters and ether moieties)—was used as flocking agent, showing significant improvement of water purification compared with action of Al_2_(SO_4_)_3_ alone and synthetic anionic polyelectrolyte.

Another work describes the application of tannin-based flocculants in effective removal of metal ions such as Zn(II), Ni(II), and Cu(II) with 75% yield at low agent dose (100–150 ppm) [128].

Larch tannin modified by Mannich reaction and then by quaternization was proposed as flocculant for removing of the *Microcystis aeruginosa* cyanobacterium from water [129]. It appeared that 100% of aromatic proteins and about 80% of protein-like substances in the extracellular organic materials is removed. However, the lower effectiveness in flocculation of humic/fulvic-like compounds has been observed.

Interesting are works regarding the use of vegetable raw materials, e.g., plum stones [72] and peanut shells [152] for heavy metals ions removal from aqueous solutions or animal ones, e.g., eggshells employed in dyes removal from wastewater [153]. These works are in accordance with the ecological trend due to food waste management.

Potential application in water treatment of common vegetables and legumes was reviewed by Choy [21]. Particular attention in this work was paid to *Fabaceae* family, e.g., peanut, soybean, guar bean, green pea, etc. The efficiency of these materials in coagulation/flocculation strongly depends on optimal agent dose and pH range.

Numerous biopolymers of plant (from, e.g., banana peel and pith, cassava peel, cactus leaves, circus peels, garden cress, lentil extract, or kenaf crude) and animal (chitin and chitosan) origin, as well as those produced by microorganisms (bacteria and fungi), proposed for separation of microalgae from water, were reviewed by Ang [141]. The surface charge and morphology, molecular weight, chemical structure, and thermal properties of those agents were determined. It appeared that such green clarifying agents often exhibit better performance than classic inorganic compounds (for instance, alum).

Other authors [154,155,156] have recommended using environment friendly materials from exotic plant sources such as nirmali (*Strychnos potatorum*) seeds [157], tannin extracted from wood of trees such as *Acacia* and *Castanea* [158] or cactus species [159,160] as flocculants designed for water and wastewater treatment. Very interesting research indicates the possibility of using natural plant extracts from macerated *Moringa Oleifera, Syzygium cumini*, and *Artcarpus heterophyllus* seeds [161] that were mostly consisted of protein and carbohydrates mixtures—not completely identified chemically. Coagulation/flocculation studies were carried out on water samples taken from local drinking water sources. The results show that the plant extracts effectively remove water turbidity and are disinfecting, therefore may be a potential useful flocculants in near future. 

**Figure 13:**
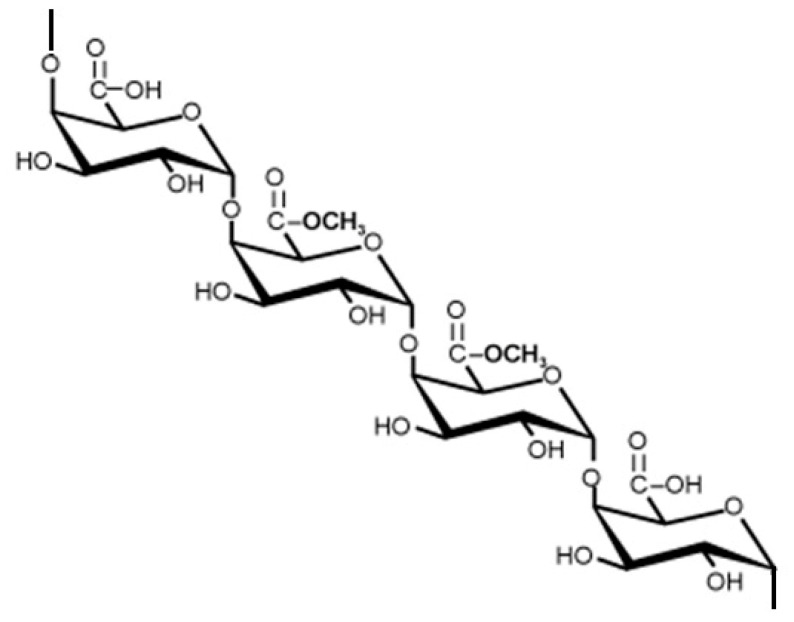
Schematic presentation of pectin [163].

Moreover, publications from recent years indicate the possibility of using pectins as valuable plant-derived flocculants [118,119,120,121,122]. Pectins are polysaccharides naturally occurring in the cell walls of fruits and vegetables, with strong gelation ability. Their structure is complex but in simplified terms it can be assumed that it consists mainly of methylated esters of polygalacturonic acid (Figure 13) [162,163,164]. 

They are linear macromolecules, but some branches with (1,2)-L-rhamnose units appear. Various functional groups, e.g., hydroxyl, carboxylic, ester, and amide, present in these polysaccharide chains can participate in hydrogen bonding and complexation leading to network formation. It must be remembered that the chemical composition of pectin may vary significantly depending on the plant source.

Flocculation activity of nopal pectins (extracted from *Opuntia ficusindica*) in removing of numerous metallic ions such as Ca^2+^, Cu^2+^, Zn^2+^, Cr^3+^, Ni^2+^, Pb^2+^, and Cd^2+^ was described by Ibarra-Rodríguez and co-workers [119]. Viscosity measurements, FTIR, Atomic Emission Spectroscopy (AES) and Scanning Electron Microscopy combined with Energy Dispersive Spectroscopy (SEM-EDS) techniques were used for characterization of the solid residue and the supernatant. The optimal dose of 0.019 mg/mL allowed for removing 99% of all metal ions.

Ho [120] found that optimum treatment of kaolin water suspension is achieved at pH 3 and pectin concentration of 3 mg/L. This dose was much lower than needed when using polyacrylamide flocculant for the same solution.

In other work [121], it was established that pectin is very effective in kaolin flocculation in the addition of small amount of Al(III) and Fe(III) ions (0.1–0.2 mM). However, in this casem the pectin dose was higher (30 mg/L). 

In Buenaño’s work [122], three natural polymer sources: green plantain peel starch, orange peel pectin, and tamarind seed extracts were the subject of research. It turned out that they obtained flocculating activity only when combined with aluminum sulfate. The high removal of turbidity (87%) and color (92%) of contaminated water was possible at relatively high ratio of aluminum sulfate to natural polymer. 

Modified pectin from citrus appeared to be useful in separation of oil and toxic Cr(VI) ions at high concentration from wastewater [118]. The modification consisted of etherification of pectin and impregnation by polyaluminum chloride. Selection of appropriate conditions of process allowed obtaining maximum efficiency of oil and chromium ion removal at level 95.0% and 98.4%, respectively.

Three types of extract from Okra (*Abelmoschus esculentus*) of various composition were considered as a potential bioflocculants [123]. The first extract consisted mainly of pectin and hemicellulose as well as mixture of other compounds such as sugars and proteins. The second one contained mainly pectins, while the third was likely hemicellulose. It was found that extract with the highest amount of pectin was able to remove ca. 70% of suspended solids from water which indicates the best bioflocculation ability of this polysaccharide among studied samples.

Owing to flocculating and suspending properties, pectin extracted from pomelo peel has been also proposed for pharmaceutical applications [165].

**Figure 14:**
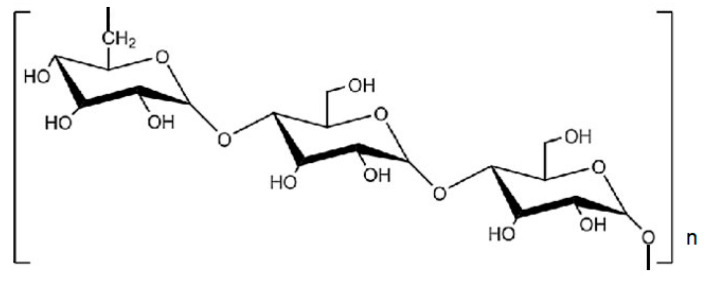
Chemical structure of pullulan.

Ghimici and co-workers analyzed the possibility of using pullulan (Figure 14) derivatives in the flocculation of pesticides [111,112] in wastewater. The modified pullulan contained either pendant tertiary amine or quaternary ammonium salts. Based on the research of UV–Vis spectroscopy, it was found that the strong interaction of pesticide particles with polycations resulted in a high degree of pollution removal (in the range of 80–98%). On the other hand, the measurements of the zeta potential allowed for the verification of the flocculation mechanism—it was mainly charge neutralization but chelation and hydrogen bonding also took place.

The spectrum of potential sources of new bioflocculants is growing every year. It can be expected that the sourcing of new polysaccharide flocculants and the development of techniques of their modification techniques will soon contribute to the displacement of conventional clarifiers based on synthetic polymers.

## 6. A New Approach in Obtaining Flocculants

### 6.1. Bioflocculants Produced by Microorganisms

Polymers synthesized by microorganisms constitute a new trend in the production of bioflocculants. These compounds are produced by selected strains of bacteria, fungi or algae naturally occurring in the sewage of various origins or in soil [166,167,168]. 

The most common bioflocculants of this type are extra-cellular polysaccharides and proteins. Increasingly, attention is paid to polymers resulting from the bacterial or fungal fermentation of carbohydrates [130]. 

The above-mentioned pullulan, obtained in the process of starch fermentation by *Aureobasidium pullulans* fungi, or xanthan resulting from the fermentation of carbohydrates by the bacteria *Xanthomonas campestris*, can be listed here [169,170]. There are many reports on the sources and methods of incubation of microorganisms used in the production of polymers showing flocculation activity [171] (Table 2). 

Great attention is focused on Extracellular Polymeric Substances (EPS), mostly exopolysaccharides, which are produced during the growth of microorganisms. They are usually complex long-chain, high-molecular-weight mixtures of macromolecules containing branched repeating units of sugars such as fructose, glucose, galactose, and mannose or their derivatives as well as non-carbohydrate organic substituents [172]. Some common examples are described below.

EPS bioflocculant polysaccharide produced by *Bacillus cereus* bacteria was tested for the removal of heavy metals from water. The ability to remediate was evaluated by measuring the reduction of bioluminescence in *Vibrio harveyi* (Gram-negative bacteria) [173]. The results show significant flocculating efficiency of obtained compound. 

As is known, the effectiveness of bioflocculants action depends on the effluent properties and process conditions such as pH, temperature, and the presence of various ions. Tang et al. [174] proposed new bioflocculant that is cation independent, pH tolerant and thermally stable. They isolated *Entherobacter sp.* ETH-2 from activated sludge and then the obtained EPS was tested for kaolin clay flocculation. This bioflocculant was characterized as a polysaccharide with hydroxyl (OH) and carboxyl (–COO–) as well as amide (–CO–NH–) functional groups. Other examples of bacterial bioflocculants are exopolysaccharides produced by halophilic bacteria growing at high salt concentration [175]. The research showed higher reduction of turbidity of treated water compared to action of conventional synthetic polymers.

Another bio-based flocculant was produced by *Bacillus sp.* bacteria [176]. It has been found to be a mixture of protein and sugar derivatives, rich in carboxyl groups, showing flocculation activity for some metal ions present in water.

Liu et al. [177] investigated *Penicillium* strains (fungi) producing compounds containing amino, hydroxyl and carboxyl groups. Owing to these groups, and also due to their relatively high molecular weight (3 × 10^5^ Da), they show good flocculation ability.

In other research, substances produced by *Pseudomonas aeruginosa* bacteria were tested in kaolin suspension [178]. These compounds identified as a mixture of proteins, carbohydrates, and their derivatives, including uronic acids, demonstrated very good flocculating properties with over 80% reduction of turbidity at a low dose (approximately 1%). 

Wang et al. [179] obtained polysaccharides from *Klebsiella mobilis* bacteria strain isolated from dairy wastewater. It was proved that such compounds have a high flocculation ability to remove some dyes from water with over 90% efficiency. 

Most important microorganisms producing EPS and their potential applications in water and wastewater treatment are listed in Table 2.

### 6.2. Nanoflocculants

Due to the development of nanotechnology in recent decades, also in the field of water purification materials of nanometric sizes are increasingly used. It is also assumed that such organic-based nanoflocculants will be characterized by longer shelf life [200].

The materials, whose particles have at least one dimension below 100 nm, exhibit different properties from their counterparts with micrometer sizes. Nanoparticles are characterized by a very developed surface, thus a very high surface area to volume ratio. This results in a large number of active points and functional groups on the surface, which positively affects the adsorption processes occurring during water treatment.

The review by Jumadi et al. [201] is devoted to recent achievements in the field of nanoflocculants. Particular attention is paid on their performance in removing of heavy metals, organic dyes, and microorganisms from water. Some considerations apply to inorganic compounds or nanocomposite flocculants (based on metal and metal oxides), which are not the subject of this work. However, among the innovative nano-sized biological materials, cellulose, chitosan, and other biomaterials discussed above can be mentioned.

Very interesting chitosan modification was shown by Mohammadi et al. [54]. Carboxylated chitosan with magnetic nanoparticles (Fe_3_O_4_) was used for nitrate, fluoride and phosphate ions removal. Studies were conducted on model aqueous solutions containing various concentrations of the above-mentioned anions. Magnetic nanoparticles, obtained by mixing of FeCl_2_ and FeCl_3_, were added to the previously prepared acidic solution of this modified polysaccharide. The results reveal that the adsorbent dose was one of the most important parameter determining the efficiency of flocculation. Increasing the amount of the polysaccharide derivative from 2 to 20 g/L allowed for gradual reduction of all ions until the equilibrium adsorption capacity was reached. Thanks to the use of magnetic nanoparticles, it was possible to completely remove agglomerates formed after the process from the aqueous solution.

Application of chitosan composite containing magnetic particles (Fe_3_O_4_) for water purification was also presented in article by Zhang et al. [202]. They grafted chitosan with methyl methacrylate, acrylic acid or 2-methylacryloyloxyethyl thrimethyl anmmonium chloride and then coated the surface of the magnetic particles. Obtained core–brush copolymers with magnetic core were applied as adsorbents to removal of pharmaceuticals such as diclofenac sodium tetracycline hydrochloride from water solution. High removal efficiencies were found owing to particular topology and enhanced surface area of copolymers. Studies of mechanism revealed that ion attraction between the positively charged polymer brushes and the anionic medicines (active substances) was the main driving force. After adsorption, the coating copolymer changes the conformation—the extended branches collapsed. The advantage of this solution is the ability to remove adsorbed impurities by applying a regular magnet. 

Another magnetic flocculant was described by Leshuk et al. [203]. Nanoparticles of Fe_3_O_4_@SiO_2_ coated with few various polymers such as poly(diallyldimethylammonium chloride), poly(sodium 4-styrenesulfonate), poly(vinylpyrrolidone), poly(acrylic acid), and chitosan were applied as magnetic agents in removing of Au, Ag, Pd, Pt, and TiO_2_ from aqueous suspensions. Formed magnetic flocks are easily separated. Furthermore, the flocculant can be recovered and reused, which is its additional advantage.

A three-component magnetic flocculant was proposed by Wang and others [204]. Nanoparticles of iron (II,III) oxide, prepared in co-precipitation in the presence of chitosan, were implemented to cellulose or biological carbon (biochar). The source of cellulose was the local plant—calamus. Biochar has been obtained in the pyrolysis of these plants. The flocculants, designed for coal slime water treatment, were characterized using the FTIR, XRD and SEM methods. It has been shown that water turbidity was reduced by ~97% and ~94%, while COD removal was ~78 and ~74% in the presence of Fe_3_O_4_-chitosan-cellulose and Fe_3_O_4_-chitosan-biochar, respectively.

Lignin, despite its renewable nature, was previously underrated as a flocking agent, but is now proving to be a promising nanomaterial for removing of microbial impurities. An innovative approach to obtaining lignin nanoparticles from switchgrass was described by Yin et al. [205]. For this purpose, lignin was treated with ultrasound in an alkaline medium and then complexed with gelatin. This novel agent was applied for removing of Gram-positive (*Staphylococcus aureus*) and Gram-negative (*Escherichia* *coli*) bacteria from wastewater. The gelatin complex with lignin nanoparticles proved to be a very effective in an acidic environment in a short time of action (90–95% yield during 30–60 min, at pH 4.5 and 5.0, respectively) for both bacteria strains. 

As mentioned above, cellulose can exist in the form of nanofibrils (CNF) or nanocrystals (CNC), which is a valuable material for obtaining flocculants [98,99,100,101,102]. Numerous works have been devoted to this topic [206,207,208,209,210,211]. 

Nanocellulose can be prepared in process of mechanical disintegration, biological and chemical treatment [206]. Nano-sized cellulosic materials found application in environmental remediation as flocculants, adsorbents, membranes, and constituents of composites. They are particularly effective in removing inorganic ions (heavy metal cations and sulfates or phosphates anions), organic dyes (e.g., Methylene Blue, Congo Red, Crystal Violet, and Malachite Green), and antibiotics from water.

To improve flocculation performance cellulose nanocrystals surface can be modified to cationic structure [211]. In purification of water from silica with flocculant obtained by CNC grafting with 3-chloro-2-hydroxypropyltrimethylammonium chloride, the turbidity was reduced by 99.7% at very low concentration of this cationic CNC (only 2 ppm).

Vandammme and coworkers [212] studied cationically modified CNC for flocculation of microalgae (Chlorella vulgaris). Two types of positively charged CNCs were obtained in esterification and nucleophilic substitution reactions. It turned out that maximal flocculation efficiency achieved even 100% at 0.1 g dose of these flocculants.

Furthermore, the effect of size of cellulosic nanomaterial on microalgae flocculation was demonstrated [213]. It was found that these microorganisms were trapped in the CNF network bound by hydrogen bonds. Moreover, they were able to grow in this network, which can be used in biodiesel synthesis.

An attempt to obtain a magnetic flocculant based on cellulose, similarly as in the case of chitosan, was described by Hizam et al. [214]. Cellulose coated magnetic nanoparticles were obtained by polymer shell cross linking with glutaraldehyde. The obtained flocculants of various composition and structure were used to purify the wastewater from the palm oil processing. It has been shown that optimal composition was a ratio of cellulose to magnetite powder of 1:1 (g/g) with glutaraldehyde volume of 1.5 mL. The reduction in turbidity, color, total suspended solid (TSS), and chemical oxygen demand (COD) was about 74.60%, 63.90%, 77.20%, and 55.80%, respectively.

To another issue was devoted work by Raj et al. [215]. In this work, fibrous nanocellulose was flocculated with two polyelectrolytes: linear cationic polyacrylamide and branched polyethylenimine, that differed in morphology, charge density, molecular weight, and polydispersity. The flocculation mechanism has been explained at the nano- and microscale by means of zeta potential, gel point, polyelectrolyte adsorption, and focused beam reflectance measurement.

The novel amphiphilic nanoflocculants based on oxidized sodium alginate has been synthesized through a conjugation of dodecylamine [106]. Material characterization by FTIR, ^1^HNMR, TGA, and Elemental Analysis (EA) allowed determining the structure of nanomicelles formed in self-assembly process in water. They took rod-like shape of size about 100 nm. Flocculating capacities were tested on the example of selected impurities: Pb^2+^ ions and bisphenol A (BPA) at different conditions. Moreover, mechanism and kinetics of process has been studied in detail using additionally XPS and adsorption isotherm. Lead ions were mainly complexed by OH and COOH groups of alginate, whereas BPA combined with dodecyl chains via hydrophobic interactions. It was found that the lead ions were adsorbed according to the Langmuir single-layer model, while the Freundlich multi-layer adsorption model applies for organic compound. The removal degree was 97.20% and 88.66% for Pb^2+^ and bisphenol A, respectively. 

The possibility of improving the efficiency of wastewater treatment using combinations of nanoflocculation and photochemical catalysis has recently been signaled [216]. In this case, the titanium dioxide was used as photocatalysts.

It is also necessary to mention nano-sized carbon, mainly carbon nanotubes (CNTs), which can be used as flocking agent. The adsorptive properties of activated carbon are well known [217,218], but recently many reports have highlighted the benefits of using CNT in water purification [219,220,221].

Activated carbon is usually obtained from plant sources (e.g., coconut shells, bamboo, peat, wood) in a simple carbonization process. However, obtaining CNTs is a more complicated and requires the use of appropriate technology, such as chemical vapor deposition, arc discharge, or laser ablation [222,223].

Simate carried out research to check whether carbon nanotubes (CNTs) can be used as heterogeneous coagulants and/or flocculants in the pretreatment of brewery wastewater [224]. A series of experiments were conducted in which the efficiencies of pristine and functionalized CNTs were compared with that of traditional ferric chloride. Turbidity, chemical oxygen demand (COD), and zeta potential measurements were used to monitor the progress of the coagulation/flocculation process. Although both types of CNTs demonstrated the ability to efficiently coagulate colloidal particles in the brewery effluent, iron chloride proved to be a better agent.

Development of new flocculants based on carbon and non-carbon nanomaterials (e.g., dendrimers, zeolites, hyperbranched polymers, or graphite oxide) have also been discussed in works on desalination of sea water and treatment of surface and groundwater [225,226,227].

Advanced nanomaterials are proposed for electrochemical flocculation (electrocoagulation) which is applied not only for water purification but also for water splitting for hydrogen production [228,229]. This electrochemical purification process consists in the destabilizing suspended, emulsified, or dissolved contaminants in an aqueous medium by an electric current. An example of such material designed for electrode is iron encapsulated in nitrogen-doped carbon nanotubes, described in detail by Yu and coworkers [228].

Although carbon nanotubes have promising flocculating properties, they have not yet found practical application in water purification and are still under intensive research.

Another class of nanomaterials proposed as flocculants are nanocomposites, which consist of a polymer matrix containing dispersed modifier particles of nanometric dimensions. Most of the literature presents, however, nanocomposite flocculants based on synthetic polymers [230,231].

An example of nanocomposite with using both synthetic and natural polymer is mentioned earlier graft copolymer of polyacrylamide/xanthan gum with silica nanoparticles [113,114]

Another nanocomposite of polyacrylamide grafted on guar gum was developed by Pal et al. [232]. During synthesis induced by microwave irradiation, silica nanoparticles have been implemented on copolymer surface which led to exceptional flocculation properties of the material due to the synergetic effect of nanosilica filler and modified guar gum.

Novel nanocomposites of polyacrylamide-grafted starch copolymers with carbon nanotubes were recently obtained in-situ method and characterized by various instrumental techniques (FTIR, TGA, DSC) [233]. On the basis of the turbidity tests and sludge volume, good flocculation efficiency in removing kaolin from the aqueous suspension has been demonstrated.

It has been proved that removal of heavy metal ions (Pb^2+^, Cr^6+^, and Ni^2+^) from mine effluents is possible using hydrogel flocculant obtained from gum karaya-grafted poly(acrylamide-co-acrylic acid) additionally containing magnetic nanoparticles of iron oxide [234].

Despite the undoubted advantages of using nanoflocculants, there are also some environmental hazards that are not yet fully identified. As in the case of using nanomaterials and nanocomposites in other areas, there are also concerns whether nanoparticles entering the environment will not cause toxic effects in nature.

### 6.3. Smart Flocculants—Stimuli Responsive Biopolymers

Smart polymers (also called stimuli responsive, stimuli sensitive, intelligent, or functional) are materials which change their properties under the influence of external stimuli, mainly change of temperature or pH, action of mechanical force, light as well as electrical and magnetic fields [235]. Under the influence of an external impulse, the polymer may alter its phase, shape, motion, functionality, and microstructure. Changes in molecular interactions in the polymer solution can lead to a phase transition, e.g., reversible gelling accompanied by contraction and expansion (swelling/de-swelling). The pH sensitivity of polymers is due to the presence of acidic or basic functional groups in the polymer chain. Therefore, future use of such materials may encompass many branches of technology and industry including water treatment [236].

Smart materials, already in use in biomedicine, are potential candidates for applications in flocculation processes, according to the current literature [28,237,238].

Although thus far synthetic polymers (e.g., derivatives of polyacrylamides, polyesters, and polyacrylates) dominate in this group of materials [28], the current trend is also the search and study of biopolymers with intelligent features [239]. 

The well-known pH stimuli biopolymer is chitosan due to the presence of amino groups, which are reversibly protonated and deprotonated dependently on the environment [137]. Other natural pH sensitive polymers are hyaluronic acid, alginic acid, and guar gum [240].

The neutral polysaccharides can be chemically modified for this purpose, e.g., grafting with acrylic acid or methacrylic acid what sensitizes them to pH changes [238,240,241]. Such polycarboxylic derivatives have different topologies: dendrimers, brushes, combs, vesicles, micelles, gels, and nanospheres. Among the materials of a polybasic nature, one can mention the (meth)acrylates, (meth)acrylamides, and vinylic polymers containing tertiary amine, morpholino, pyrrolidine, imidazole, piperazine, and pyridine groups, which can also be used for chemical modification of biopolymers.

Thermo-responsive cellulose ether, synthesized by grafting of butyl glycidyl ether onto hydroxyethyl cellulose, turned out to be effective in removing of organic dye (Nile Red) from wastewater [242]. An additional advantage is that this flocculant can be easily recycled and reused.

Another smart flocculant containing biomaterial has been synthesized and characterized in Kiran’s group [243]. In the first stage, N-isopropylacrylamide/di-methylacrylamide di-block copolymer was obtained in reversible addition–fragmentation chain transfer-mediated polymerization (RAFT). In the second stage, this thermo-responsive copolymer has been grafted onto β-cyclodextrin to make biodegradable material. This intelligent and ecofriendly flocculant shows good separation of kaolin from aqueous suspension. 

Kocak and coworkers collected information about pH responsive polymers [240]. Besides synthetic functionalized polymers, they also cited appropriately modified biopolymers, namely cellulose. Moreover, the possibility of producing pH sensitive materials from polypeptides such as poly(l-glutamic acid), poly(histidine), and poly(aspartic acid) is also mentioned.

Lemanowicz et al. considered the effect of stimuli-responsive polymers in stabilization/destabilization of solid particles dispersed in aqueous solutions [32]. Mechanistic consideration of such flocculation process concerns mainly synthetic polymeric agents. 

Although relatively much attention in the literature is devoted to adsorbents based on stimuli sensitive biopolymers, which can be used for water and wastewater treatment [238,244,245,246], flocculants of this type are rarely presented, although their importance is emphasized. In our opinion, these are prospective materials that require further extensive research.

## 7. Conclusions 

Flocculants found applications in various types of technological processes that require purification of water from different types of suspended particles (inorganic, organic, and microbial). They are used, among others, in the dairy industry, petroleum industry, mining, metallurgy, papermaking, and in the treatment of drinking water and municipal sewage [5,30,247,248]. Currently, many scientific works are devoted to obtaining bioflocculants of plant or, less frequently, animal origin. Recent studies focus on the use of readily available, safe, and cheap biopolymers (e.g., polysaccharides), which are biodegradable. To improve their flocculation efficiency, polysaccharides are subjected to chemical modification (e.g., graft copolymerization with synthetic monomers) or by physical mixing with inorganic agents Biomaterials obtained by biosynthesis in the presence of microorganisms also show promising properties. Other modern types of flocculants are nanomaterials (e.g., polymer nanocomposites) or stimuli-responsive, i.e., intelligent, flocculants, which seem to be materials of the future.

The mechanism of flocculation with biopolymers is relatively well known but not fully understood. The various factors (e.g., pH, ionic strength or shear rate, impurities concentration, and flocculant dose) have a significant impact on the course of the process. As this literature review shows, biopolymers have a great potential to become effective flocculating agents for water purification, but so far they are not used on a large scale in industrial practice. The main direction of future research is the acquisition of new biomaterials and their modification in order to optimize the flocculation process.

## Figures and Tables

**Table 1:** Polysaccharide flocculants used in water and wastewater treatment.

Bio-Based Flocculants	Applications	Reference
**Starch:**		
**Cationic starch**	Turbidity removal from suspension of kaolin, bentonite, and natural clay	[82,83]
**CMS-CTA** ((2-hydroxypropyl) trimethylammonium chloride etherified carboxymethyl starch	Clarification of kaolin and hematite suspension	[84]
**DCS** (dispersible cationic starch)	Kaolin suspensions clarification	[85]
**St-g-PAM** (polyacrylamide grafted starch)	Kaolin suspension clarification	[16]
**HES-g-Poly-(DMA-co-AM)** (hydroxyethyl starch grafted poly-(N,N-dimethylacrylamide-co-acrylamide)	Removal of metal ions	[86]
**HES-g-Poly-(DMA-co-AA)** (hydroxyethyl starch grafted poly-(N,N-dimethylacrylamide-co-acrylic acid)	Removal of dye from its aqueous solution	[87]
**Chitosan:**		
**Chitosan solution**	Surface water treatment	[88]
**Chitosan solution**	Turbidity and TDS removal	[89]
**CMC-g-PAM** (carboxymethyl chitosan-graft-polyacrylamide)	Dyes removal from aqueous solutions	[17]
**CAMFA** (chitosan-acrylamide-fulvic acid)	Color removal	[37]
**CMC-g-PAM** (carboxymethyl chitosan-g-polyacrylamide)	Dyes removal	[90]
**CMC-g-PDMC** (carboxymethyl chitosan-g-poly(2-methacryloyloxyethyl) trimethyl ammonium chloride)	Dyes removal	[91]
**CMC-CTA** (amphoteric carboxymethyl chitosan)	Turbidity removal	[92]
**CTS-g-PAA** (chitosan grafted copolymer of acrylamide and 3-acrylamide propyltrimethylammonium chloride)	Dye removal	[69]
**Carboxylated chitosan/Fe_3_O_4_**	Removal of fluoride, nitrate and phosphate from aqueous solution	[54]
**Chitosan**		
**CAC** (carboxylated chitosan-graft-polyacrylamide- co-sodium xanthate);	Turbidity removal	[93]
**CPCTS-g-P(AM-AMPS)** (carboxylated chitosan-graft-poly[acrylamide-2-acrylamido-2-methylpropane sulfonic acid])	Heavy metal removal	[94]
**CS-g-PAD** (chitosan-g-poly(acrylamide- acryloyloxyethyl) trimethylammonium chloride)	Water purification from zinc phosphate	[95]
**Cellulose:**		
**Cationic cellulose**	Water decolorization	[79]
**HPMC-g-PAM** (hydroxypropyl methyl cellulose grafted with polyacrylamide)	Clarification of kaolin and iron-ore suspension	[96]
**CMCNa** (sodium carboxymethyl cellulose)	Turbidity removal from drinking water	[97]
**DCC** (dicarboxylic acid nanocellulse)	Municipal wastewater treatment; Turbidity removal	[98,99]
**Carboxymethyl cellulose-g-polyacrylamide**	Kaolin suspension clarification	[18]
**CCNF** (cationic cellulose nano-fibers)	Flocculation in pulp slurries	[100]
**PAETMAC-g-CNC** (polyacryloyloxyethyltrimethyl ammonium chloride-g-cellulose nanocrystal)	Decolorization of colored effluents	[101]
**ADAC** (three anionic sulfonated nanocellulose)	Turbidity and COD removal	[102]
**QC** (water-soluble quaternized cellulose)	Anionic dyes solution remediation	[103]
**Anionic and cationic cellulose**	Textile industry effluent treatment	[49]
**MCC(pAA-co-pDMC)** (grafted microcrystalline cellulose)	Decolorization and turbidity removal	[104]
**Cellulose fibers, membranes, aerogels and chemically modified cellulose materials**	Treatment of water contaminated by oil spills (removal of organic and inorganic matter, adsorption of heavy metals)	[105]
**Alginate**		
**DDA-conjug-alginate**	Removal heavy metal ions and organic pollutants from wastewater	[106]
**SAG-g-NVP** (sodium alginate-g-N-vinyl-2- pyrrolidone)	Coal fine suspension clarification	[107]
**SA-CTA** (sodium alginate ampfoteric derivative)	Heavy metal ions and humic acids removal	[108]
**SAD** (sodium alginate-dimethyl diallylammonium chloride)	Water decolorization	[109]
**Pullulan**		
**P-g-pNIPAAm** (pullulan-g-p(N-isopropyl-acrylamide)	Turbidity removal	[110]
**Cationic Pullulan**	Removal of pesticides	[111,112]
**Xanthan gum**		
**XG-g-PAM/SiO_2_** (polyacrylamide grafted xanthan gum/silica hybrid nanocomposite)	Mine wastewater treatment for color removal, treatment of synthetic effluents and removal of Pb(II) ions from aqueous solution	[113,114]
**Xanthan-g-PDMA** (xanthan grafted N,N-dimethylacrylamide)	Wastewater treatment	[115]
**Dextran**		
**Ionized dextrans Dex-AM-AS** (dextran-g-poly(acrylamide-co-sodium acrylate)	Removal of turbidity and pesticides	[116]
**DAB** (dextran-g-bezyl(methacrylooyloxyethyl)dimethylammonium chloride)	Removal dyes from wastewater	[117]
**Pectin**		
**Etherified pectin and polyalluminium chloride**	Removal of oil and Cr(VI) from wastewater	[118]
**Nopal pectin**	Heavy metal ions removal	[119]
**Citrus pectin**	Kaolin suspension treatment	[120]
**Apples pectin**	Kaolin suspension treatment	[121]
**Orange peel pectin**	Turbidity removal	[122]
**Okra extracts**	Suspended solids removal	[123]
**Guar gum**		
**HPTAC-guar** (hydroxyl-propyl triammonium chloride guar gum)	Removal of COD, turbidity and biological contaminants from municipal wastewater	[124]
**CGG** (cationized guar gum)	Bentonite aggregation	[125]
**Lignin and tannin**		
**OSKL** (sulfomethylated softwood kraft lignin)	Removing of cationic dye	[50]
**KLD** (kraft lignin copolymer)	Turbidity removal	[126]
**Tannin**	Turbidity removal	[127]
**Tanfloc** (vegetal water-extracted tannin)	Heavy metal removal	[128]
**A-TN, Q-TN** (chemically modified larch tannin and its quaternized derivative)	Algal water treatment	[129]

**Table 2:** Examples of microorganism for bioflocculant production and their potential applications in water and wastewater treatment.

Microorganism	Applications	Reference
***Bacillus cereus***	Wastewater treatment for heavy metal removal	[173]
***Enterobacter* sp. **	Kaolin clay flocculation	[174]
***Klebsiella* sp. **	Water treatment; removal of amoeba cyst from water; sludge dewatering	[172,180,181]
***Mucor rouxii***	Wastewater treatment	[182]
***Achromobacter* sp. **	Wastewater treatment	[183]
***Bacillus and Streptomyces* sp. **	Swine wastewater treatment	[184]
***Bacillus and Rhizobium radiobacter***	Water treatment	[185]
***Basillus* sp. **	Treatment of wastewater	[186]
	Treatment of low temperature drinking water	[187]
	Industrial wastewater treatment (COD removal and dye decolorization)	[188]
***Penicillium* sp. **	Management of industrial wastewater	[188]
***Herbaspirillium* spp. *and Pseudomonas* sp. **	Industrial effluents and wastewater treatment (suspension particle and heavy metals removal)	[189]
***Rhodococcus* sp. **	Treatment of swine wastewater	[190]
***Serratia* sp. **	Treatment of wastewater	[191]
***Staphylococcus and Pseudomonas* sp. **	Treatment of industrial wastewater (COD, indigotin and dyeing wastewater)	[192]
***Proteus mirabilis***	Wastewater treatment (waste sludge dewatering)	[193]
***Aspergillus flavus***	Suspended solids removal	[194]
***Klebsiella variicola***	Removal of turbidity and SS in drinking water	[195]
***Bacillus firmus***	Water treatment (removal of metal ions such as Pb, Cu, Zn)	[196]
***Trichoderma* sp. **	Heavy metals ions removal	[197]
***Streptomyces platensis***	Kaolin clay flocculation	[198]
***Oceanobacillus polygoni***	Tannery wastewater treatment	[199]
